# Plasma Metabolomics Reveals a Shared Metabolomic Profile in Experimental and Human Chronic Kidney Disease

**DOI:** 10.3390/toxins18050225

**Published:** 2026-05-09

**Authors:** Søren H. Elsborg, Jasmine C. L. Atay, Johan Palmfeldt, Christian Daugaard Peters, Krista Dybtved Kjærgaard, Henricus A. M. Mutsaers, Rikke Nørregaard

**Affiliations:** 1 Department of Clinical Medicine, Aarhus University, 8200 Aarhus N, Denmark; sohe@biomed.au.dk (S.H.E.); jcla@clin.au.dk (J.C.L.A.); h.a.m.mutsaers@clin.au.dk (H.A.M.M.); 2 Research Unit for Molecular Medicine, Department of Clinical Medicine, Aarhus University, 8200 Aarhus N, Denmark; johan.palmfeldt@clin.au.dk; 3 Department of Renal Medicine, Aarhus University Hospital, 8200 Aarhus N, Denmark; chipte@rm.dk (C.D.P.); krista.kjaergaard@rm.dk (K.D.K.)

**Keywords:** chronic kidney disease, metabolomics, biomarkers, uremic toxins

## Abstract

Chronic kidney disease (CKD) affects nearly 10% of the global population, yet diagnosis and disease monitoring still rely primarily on plasma creatinine. Because creatinine levels are strongly influenced by non–renal factors, such as age, sex, muscle mass, and diet, its accuracy as a kidney function marker is limited. To identify plasma biomarkers that reflect kidney injury, we applied untargeted and targeted metabolomics in the adenine-induced CKD mouse model, a well-known tubular damage model, and validated the findings in plasma from patients with advanced CKD and healthy controls. We identified five metabolites that showed altered plasma levels in both experimental and human CKD, of which galactonic acid, pipecolic acid, and N-acetylneuraminic acid were significantly associated with measured glomerular filtration rate (GFR). As a proof-of-concept, we demonstrated that integrating these metabolites into a biomarker panel alongside creatinine could improve GFR estimation compared with creatinine alone. Our study introduces a promising metabolite-based biomarker panel that might enhance the accuracy of kidney function assessment and could potentially support diagnosis, risk stratification, and monitoring of disease progression; however, validation in a broader CKD cohort is needed.

## 1. Introduction

Chronic kidney disease (CKD) affects at least one in ten people globally over the course of their lifetime and is a main contributor to mortality. In 2021, CKD was the seventh leading cause of death worldwide [1,2] and is projected to become the fifth leading cause of death by 2040 [3]. Approximately 15% of patients with CKD progress to end-stage kidney disease within ten years [4], requiring life-saving renal replacement therapies, such as dialysis or kidney transplantation [5].

In clinical practice, the diagnosis of CKD is primarily based on the glomerular filtration rate (GFR), which is the most important functional measure of kidney health. Despite the central role of GFR in the management of CKD, direct measurement of GFR is difficult and time-consuming in the clinic, which is why it is most often estimated from plasma creatinine levels [6,7]. However, plasma creatinine is affected by various factors unrelated to kidney function, including age, sex, muscle mass, and diet [7,8]. In addition, it is a poor indicator of early histological kidney damage, often failing to reflect ongoing pathological changes at early CKD stages [9]. Thus, there is an urgent need for more accurate markers that are associated with kidney damage.

To improve assessment of renal function, urine albumin-to-creatinine ratio and an alternative marker of GFR based on plasma Cystatin C, have been introduced into clinical practice [7,10]. However, these markers mainly reflect glomerular permeability and filtration function and provide limited insight into the presence of interstitial fibrosis and tubular injury, which are key features of CKD [8,11]. Importantly, a growing body of evidence indicates that tubulointerstitial damage, particularly interstitial fibrosis and tubular atrophy, is a stronger predictor of renal function decline than glomerular injury [12,13]. Consequently, biomarker discovery has shifted towards identifying markers of tubulointerstitial damage. Recently, untargeted plasma metabolomics has emerged as a powerful approach capable of detecting small metabolites and solutes that accumulate in the plasma of CKD patients, both at the early and late stages of the disease [14,15].

Animal models are instrumental for elucidating the pathophysiological mechanisms of CKD and for identifying potential biomarkers [16]. However, to identify clinically relevant biomarkers, models that accurately reflect the onset and progression of human CKD are needed. For example, the Adriamycin model is relevant for studying glomerular injury [16], whereas the 5/6 nephrectomy model is suitable for investigating late-stage CKD by replicating extensive nephron loss [16]. The adenine-induced CKD model is useful for studying tubular damage and the associated plasma accumulation of metabolic waste products [17]. This model is based on the enzymatic conversion of exogenously administered adenine into 2,8-dihydroxyadenine, which forms crystals in the renal tubular lumen, triggering tubulointerstitial inflammation, fibrosis, and loss of function [18,19]. This makes the adenine model particularly suitable for identifying biomarkers of tubular injury. In addition, the model is well-suited to study the accumulation of metabolic waste products, including uremic toxins [20].

In this study, we employed untargeted metabolomics to identify candidate markers of renal injury in mice with adenine-induced CKD. This approach minimized the influence of confounding factors that often complicate biomarker studies in CKD patients. To enhance the translational relevance of our findings, we validated the identified metabolites in a cohort of patients with advanced CKD and healthy controls and, as proof-of-concept, assessed their potential to estimate GFR.

## 2. Results

### 2.1. Adenine-Feeding Reduces Kidney Function and Induces Tubulointerstitial Injury

**Figure 1:**
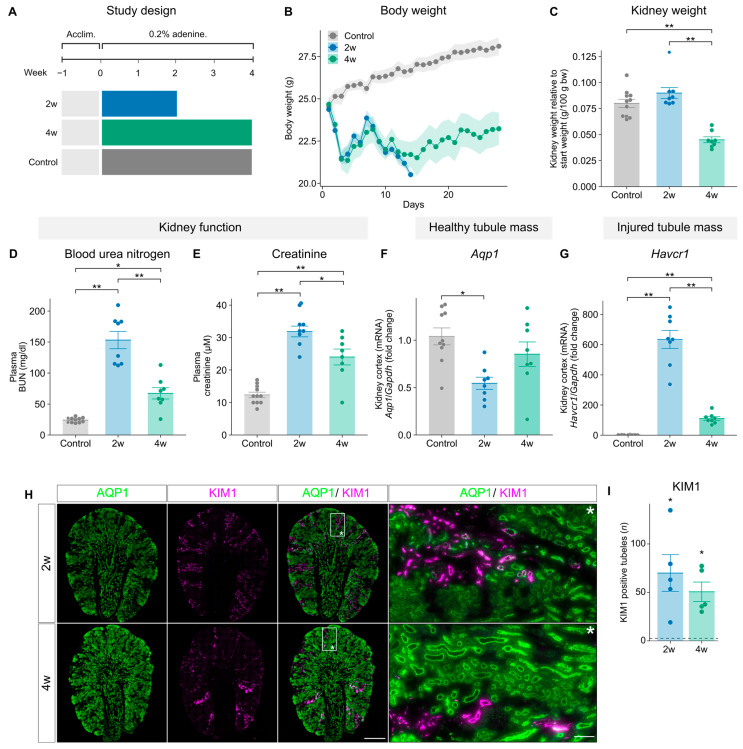
Kidney dysfunction and tubular injury in mice induced by 0.2% dietary adenine. (**A**) Study design: C57BL/6NRj male mice were fed a 0.2% adenine-enriched diet for 2 weeks (*n* = 8) or 4 weeks (*n* = 8) or the same diet without adenine (*n* = 11). (**B**) Changes in body mass during the intervention period (2-week and 4-week groups; *n* = 8 and controls; *n* = 11). (**C**) Left kidney weight was measured at the end of the study and expressed relative to start body weight (2-week and 4-week groups; *n* = 8 and controls; *n* = 11). (**D**,**E**) Blood urea nitrogen (BUN) and plasma creatinine concentrations were measured at study end as markers of kidney function (2-week and 4-week groups; *n* = 8 and controls; *n* = 11). (**F**,**G**) Kidney cortex mRNA expression levels of *Aqp1* (marker of healthy tubules) and *Havcr1* (marker of injured tubules) were assessed by qPCR, normalized for *Gapdh* expression and the mean ΔCt of the controls, and presented as fold change (2-week and 4-week groups; *n* = 8, controls; *n* = 10). (**H**) Immunofluorescence staining for aquaporin 1 (AQP1) (green) and kidney injury molecule 1 (KIM1) (magenta) of whole kidney sections mounted on coverslips in the 2-week group (upper panels) and 4-week group (lower panels) of adenine-feeding, showing healthy (AQP1-positive) and injured (KIM1-positive) tubules. Inserts show magnified regions (white rectangles); representative images are shown (*n* = 5); asterisks indicate the orientation; left scale bar = 1 mm; right scale bar = 100 µm. Images were acquired on a Nikon Eclipse Ti2 microscope ((Tokyo, Japan) (60× oil objective). (**I**) Quantification of KIM1-positive tubules in whole-kidney sections (*n* = 5). (**A**–**H**) Data from the control and 4-week groups have previously been published [23]. Data are presented as mean ± SEM. Statistical significance was assessed using one-way ANOVA followed by the Tukey–Kramer post hoc test or Kruskal–Wallis test as appropriate; *: *p* < 0.05; **: *p* < 0.001.

**Figure 2:**
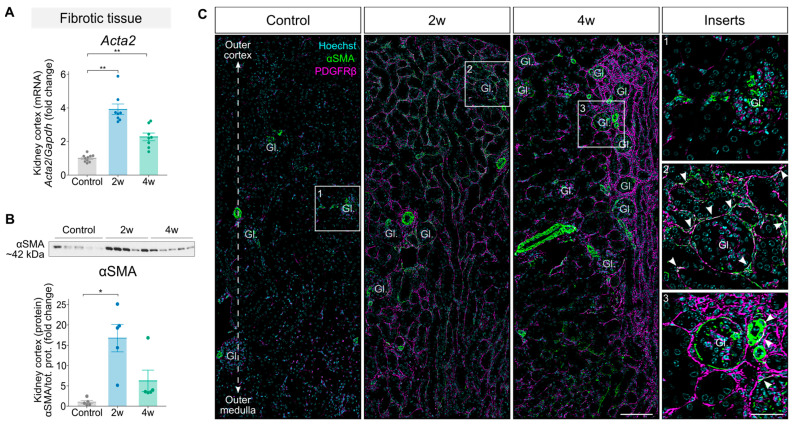
Interstitial fibrosis in mice induced by 0.2% dietary adenine. (**A**) Kidney cortex mRNA expression levels *Acta2* (marker of myofibroblasts) were assessed by qPCR, normalized for *Gapdh* expression and the mean ΔCt of the controls, and presented as a fold change (2-week and 4-week groups; *n* = 8, controls; *n* = 10). (**B**) Kidney cortex protein expression levels of alpha smooth muscle actin (αSMA) were assessed by Western blotting and normalized to total protein intensity and expressed as fold change compared to control levels (*n* = 5). (**C**) Immunofluorescence staining of Hoechst (marks the nuclei, cyan), αSMA (marker of myofibroblasts, green), and PDGFRβ (marker of fibroblasts, magenta) in the control, 2-week and 4-week groups. The images show the outer cortex (top) and outer medulla (bottom); inserts show magnified regions (white rectangles); white arrowheads point at overlapping staining, indicating activated myofibroblasts; representative images are shown (*n* = 5); left scale bar = 100 µm; right scale bar = 40 µm. Images were acquired on an Olympus VS120 Virtual Slide Scanner (Tokyo, Japan) (40× air objective). (**A**–**C**) Data from the controls and 4-week group have previously been published [23]. Data are presented as mean ± SEM. Statistical significance was assessed using the Kruskal–Wallis test; *: *p* < 0.05; **: *p* < 0.001.

We subjected C57BL/6NRj male mice to a diet enriched with 0.2% adenine [18]. The mice received the diet for either 2 or 4 weeks to mimic different kidney disease phenotypes, while control mice received the same diet without adenine (Figure 1A). The adenine-fed mice exhibited significant weight loss over the course of the study (Figure 1B), consistent with previous reports [20,21]. In the 4-week group, kidney weight was significantly reduced (Figure 1C) alongside an increase in fibrotic area as revealed by H&E staining (Supplemental Figure S1A). Furthermore, adenine-feeding resulted in an impairment of kidney function, as shown by elevated blood urea nitrogen (BUN) and plasma creatinine levels in both the 2- and 4-week groups compared to controls (Figure 1D,E), with the highest levels observed in the 2-week group. Next, we investigated the expression of markers related to proximal tubule (PT) health and injury, as the adenine model primarily induces kidney tubulointerstitial injury [18]. Expression levels of *Aqp1*, a marker of healthy PT cells, were significantly reduced in the 2-week group but not in the 4-week group (Figure 1F). The expression levels of *Havcr1* (encoding kidney injury molecule 1 [KIM1]), a marker of injured PT cells, were significantly increased at both time points compared to controls, with a significantly higher expression in the 2-week group compared to the 4-week group (Figure 1G). Quantification of KIM1-positive tubules confirmed increased KIM1-expression in the tubules from both the 2-week and 4-week groups (Figure 1I). To strengthen these findings, we performed aquaporin 1 (AQP1) and KIM1 co-staining. This revealed an inverse staining pattern, where AQP1-negative tubules were positive for KIM1 and vice versa, which was evident in both adenine-fed groups (Figure 1H). Control kidneys showed uniform AQP1 staining (Supplemental Figure S1B). To further characterize the extent of kidney injury, we analyzed the expression of *Acta2* (encoding alpha smooth muscle actin [αSMA]), a marker of myofibroblast activation and fibrosis. Both mRNA and protein levels were elevated in the 2- and 4-week groups, with the highest expression observed in the 2-week group (Figure 2A,B). Co-staining for αSMA and PDGFRβ, markers of activated myofibroblasts [22], revealed increased colocalization in the tubulointerstitial compartment, particularly in the 2-week group (Figure 2C and Supplemental Figure S2). The expression levels of additional inflammatory mediators (*Ccl2*, *Tnf* and *Il6*) and fibrosis markers (*Tgfb1*, *Fn1* and *Col1a1*) were elevated at both time points (Supplemental Figure S3A–F). Together, these findings demonstrate that a 0.2% adenine diet in the 2- and 4-week groups induces kidney injury, characterized by a reduction in kidney function and loss of PT mass in combination with increased tubular injury, interstitial fibrosis and inflammation.

### 2.2. Untargeted Plasma Metabolomics Reveals Stage-Specific Changes in the Plasma Metabolome During Adenine-Induced Kidney Disease

**Figure 3:**
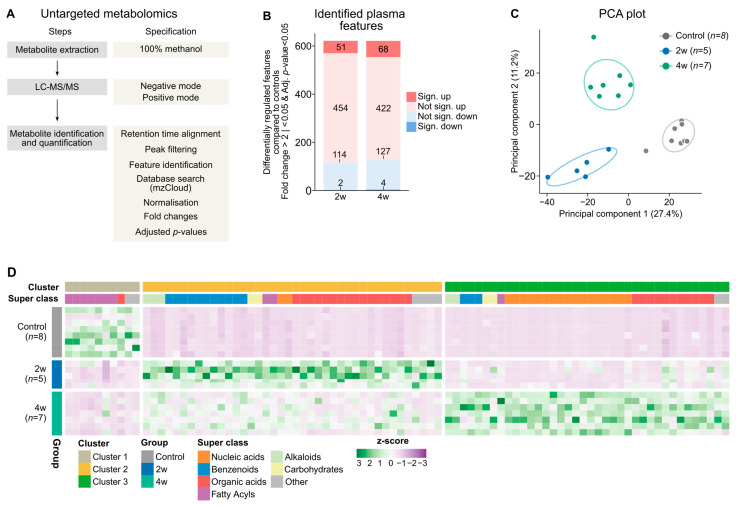
Untargeted metabolomics revealed major changes in the plasma metabolome over 2 and 4 weeks of adenine feeding. (**A**) Schematic overview of the untargeted metabolomics workflow used to analyze plasma samples from mice in the control, 2-week, and 4-week groups. (**B**) Stacked bar plot depicting up- and downregulated features in the 2-week (*n* = 5) and 4-week (*n* = 7) groups compared to the control group (*n* = 8). Significance (sign.) was defined as Benjamini–Hochberg adjusted *p* < 0.05 and fold change >2 (saturated red; significantly up) or <0.5 (saturated blue; significantly down). Non-significant (not sign.) changes are shown in pale red (increased) and pale blue (decreased). (**C**) Principal component analysis (PCA) of the plasma metabolome of the 2-week, 4-week and control groups. Principal component 1 explained 27.4% and principal component 2 explained 11.2% of the total variance in the dataset. Each point represents an individual mouse; ellipses indicate the 95% confidence interval for each group. (**D**) Heatmap of the top 90 differentially abundant plasma features across the experimental groups displayed as z-scores. Features were annotated by molecular class using RefMet, clusters were identified by k-means clustering, and the optimal number of clusters was determined using the elbow method.

We next investigated the metabolomic profile of the adenine-fed mice by performing untargeted metabolomics on plasma samples (Figure 3A). Most of the identified features (as defined by a molecule with a unique mass-to-charge ratio and retention time, i.e., the time point the feature elutes from the column) were enriched in plasma and to a similar extent in both the 2-week and 4-week groups compared to controls (Figure 3B). However, principal component analysis (PCA) revealed distinct plasma metabolome profiles across the groups, with the most pronounced separation observed between the 2-week group and controls (Figure 3C). To characterize these metabolic differences, we visualized the 90 most differentially abundant features in a heatmap and classified them by molecular class and clustering pattern (Figure 3D). Here, three distinct clusters emerged, each corresponding to one of the experimental groups. Cluster 1 was dominated by fatty acyls, which were primarily enriched in the control mice. Cluster 2 was dominated by a broader range of molecular classes, which were mainly increased in the 2-week group, but with benzenoids and organic acids as the most enriched classes. Cluster 3 contained features almost exclusively enriched in the 4-week group, most of which were nucleic acid derivatives, followed by organic acids. Together, these results indicate that adenine-induced CKD leads to a general accumulation of circulating metabolites, but the composition differed across the two time points. Fatty acyls were depleted in both adenine-fed groups. In contrast, the 2-week group exhibited a metabolite signature enriched with benzenoids and organic acids, while the 4-week group was characterized by an accumulation of nucleic acid-related metabolites.

### 2.3. Expression of Proximal Tubule Transporters Is Altered During Adenine-Induced Kidney Disease

**Figure 4:**
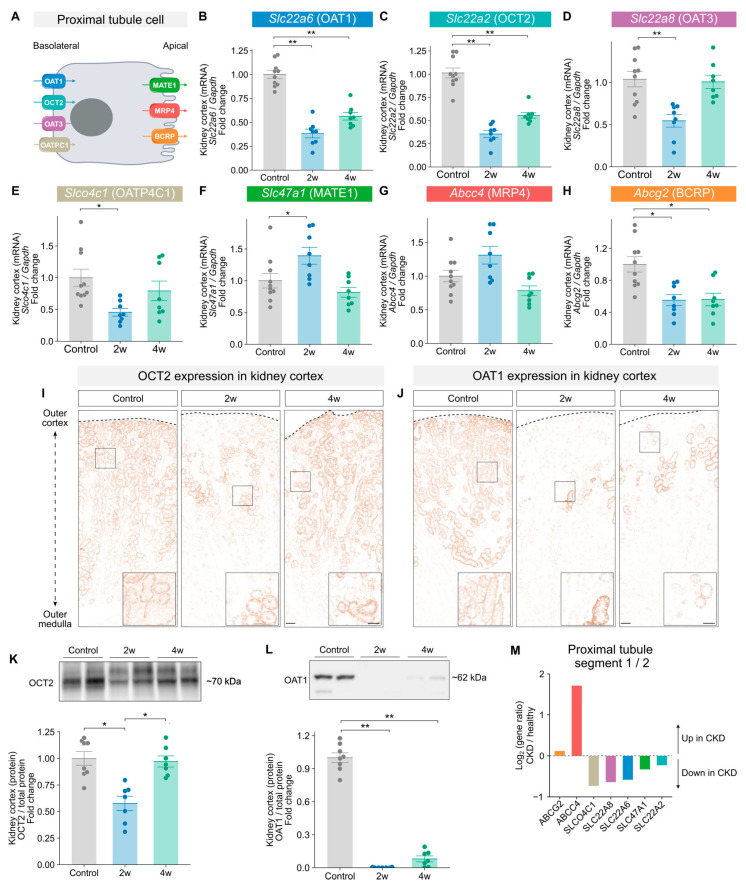
Impaired expression of proximal tubule (PT) organic compound transporters in adenine-induced CKD. (**A**) Schematic illustration of the localization and the transport direction of the investigated basolateral and apical transporters in PT cells. (**B**–**H**) Kidney cortex mRNA expression of basolateral transporters (**B**) *Slc22a6* (OAT1), (**C**) *Slc22a2* (OCT2), (**D**) *Slc22a8* (OAT3), (**E**) *Slco4c1* (OATP4C1) and apical transporters (**F**) *Slc47a1* (MATE1), (**G**) *Abcc4* (MRP4) and (**H**) *Abcg2* (BCRP) were determined by qPCR; expression levels in the 2-week (*n* = 8), 4-week (*n* = 8) and control groups (*n* = 11) were normalized for *Gapdh* expression and the mean ΔCt of the controls and presented as a fold change. (**I**,**K**) Kidney cortex protein expression of OCT2 (**I**) and OAT1 (**K**) was assessed by Western blotting in the 2-week (*n* = 7), 4-week (*n* = 7) and control groups (*n* = 8), normalized to total protein content, and expressed relative to the control group; a representative blot is shown. (**J**,**L**) Immunoperoxidase staining of OCT2 (**J**) and OAT1 (**L**) in kidney sections from the control (*n* = 5), 2-week (*n* = 5), and 4-week (*n* = 5) groups; images were acquired on a Nikon Eclipse Ti2 microscope (20× air objective); representative images are shown; scale bars = 400 μm; (**M**) mRNA levels of proximal tubule transporters were assessed by sc-RNA sequencing and expressed as a Log2 ratio between CKD and healthy patients. The results are based upon data generated by the Kidney Precision Medicine Project. Data are presented as mean ± SEM. Statistical significance was assessed by one-way ANOVA followed by the Tukey–Kramer post hoc test or the Kruskal–Wallis test as appropriate; *: *p* < 0.05; **: *p* < 0.001.

Next, we explored if the observed changes in the metabolic profile were concurrent with changes in clearance capacity. Therefore, we examined differences in the expression of key basolateral and apical PT transporters (Figure 4A) that are responsible for the renal excretion of organic compounds [24,25]. mRNA expression analyses revealed a downregulation of *Slc22a6* (organic anion transporter 1 [OAT1]), a major basolateral organic anion transporter, and of *Slc22a2* (organic cation transporter 2 [OCT2]), a major basolateral cation transporter, in both the 2- and 4-week groups compared to controls (Figure 4B,C). However, Western blot analysis showed a significant reduction in OCT2 protein levels only in the 2-week group (Figure 4K). This finding was confirmed by immunohistochemistry staining, which revealed fewer OCT2-positive tubules, particularly in the cortex from mice in the 2-week group (Figure 4I). In contrast, OAT1 protein levels were markedly reduced in both the 2-week and 4-week groups (Figure 4L), consistent with immunohistochemistry findings showing a near-complete loss of OAT1-positive tubules at both time points (Figure 4J). For *Slc22a8* (OAT3) and *Slco4c1* (organic anion transporting polypeptides 4C1 [OATP4C1]), two other basolateral transporters, the mRNA expression levels were significantly reduced only in the 2-week group (Figure 4D,E). In contrast, the mRNA expression of the apical transporters *Slc47a1* (multidrug and toxin extrusion protein 1 [MATE1]) and *Abcc4* (Multidrug resistance-associated protein 4 [MRP4]) was significantly increased in the 2-week group compared to controls, but not in the 4-week group (Figure 4F,G). *Abcg2* (breast cancer resistance protein [BCRP]) expression was significantly reduced in both the 2-week and 4-week groups (Figure 4H). Importantly, similar changes in transporter expression have been observed in CKD patients (Figure 4M; these results are based upon data generated by the Kidney Precision Medicine Project [26]). Previous studies have shown that these transporters also handle the excretion of uremic toxins (UTs), which are solutes known to accumulate in the circulation during CKD [27]. Therefore, we measured a panel of key UTs to assess if these also accumulate in the plasma of adenine-fed mice. Kynurenic acid and hippuric acid were significantly increased in the 2-week group compared to controls (Supplemental Figure S4F,G), while kynurenic acid, indoleacetic acid, and kynurenine were increased in the 4-week group (Supplemental Figure S4F,H,I). In contrast, tryptophan, phenylacetic acid and *p*-cresol sulfate were not significantly changed in the adenine-fed mice (Supplemental Figure S4J–L). In summary, adenine-feeding resulted in an altered expression profile of several basolateral and apical PT transporters, as well as increased plasma levels of archetypical UTs.

### 2.4. Identification of Candidate Metabolites Associated with Adenine-Induced Kidney Disease

**Figure 5:**
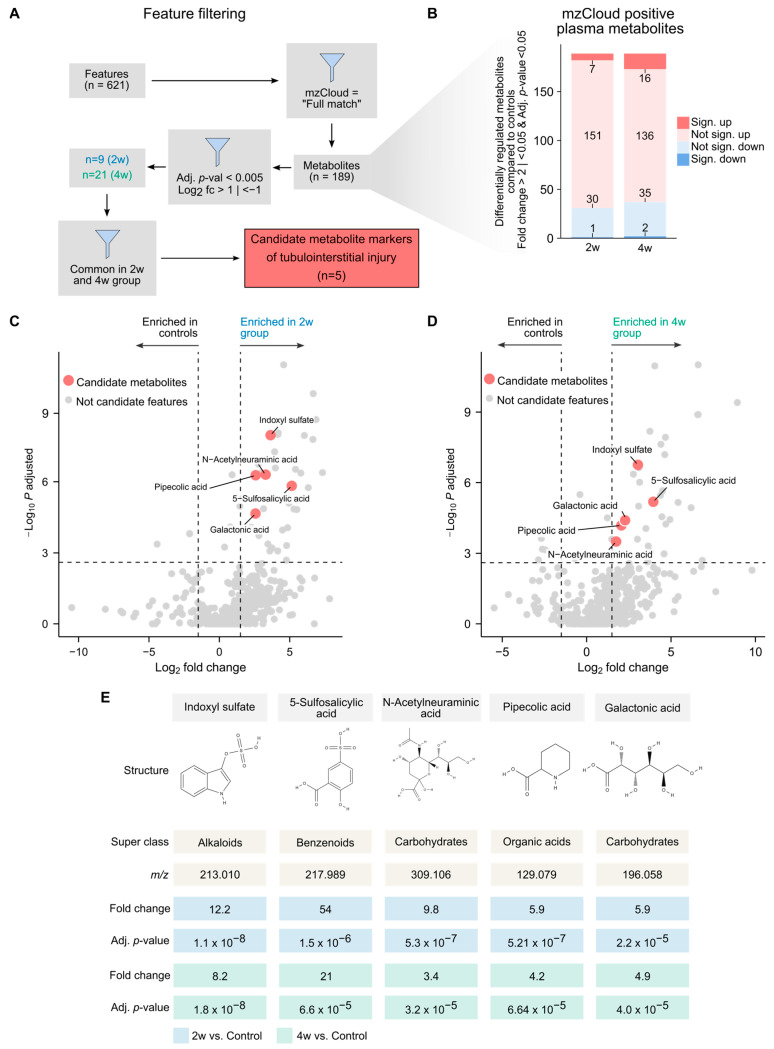
Identification and validation of candidate metabolites associated with adenine-induced CKD. (**A**) Schematic illustrating the metabolite screening workflow, narrowing down differentially abundant plasma features to top candidate metabolites associated with kidney disease after 2- and 4 weeks of adenine-feeding. (**B**) Stacked bar plot depicting up- and downregulated metabolites in the 2-week (*n* = 5) and 4-week (*n* = 7) groups compared to the control group (*n* = 8). Significance was defined as Benjamini–Hochberg adjusted *p* < 0.05 and fold change > 2 (saturated red; significantly up) or <0.5 (saturated blue; significantly down). Non-significant changes are shown in pale red (increased) and pale blue (decreased). Only features with MS/MS fragmentation spectra matched to the mzCloud database are shown. (**C**,**D**) Volcano plots showing differentially expressed plasma features in the 2-week (*n* = 5) group (**C**) and 4-week (*n* = 5) group (**D**) compared with the control group (*n* = 8). Candidate metabolites are highlighted in red. Significance was defined as Benjamini–Hochberg adjusted *p* < 0.005 and fold change > 2 or <0.5, vertical and horizontal dashed lines, respectively. (**E**) Overview of the candidate metabolites, including structural characteristics, mass-to-charge ratio (*m*/*z*), fold changes (calculated as mean peak area relative to controls), and Benjamini–Hochberg adjusted (Adj.) *p*-values.

So far, our data have shown that the adenine model is suitable for studying renal injury and the concurrent retention of plasma metabolites. We next sought to identify specific circulatory metabolite markers associated with tubulointerstitial kidney disease by comparing the metabolite levels across the 2-week and 4-week groups with the controls. To refine the candidate list, we applied stepwise filtering to reduce the number of features from hundreds to only a small subset of metabolites (Figure 5A). First, we restricted the dataset to mzCloud-confirmed positive identifications to enhance the reliability of our feature annotation, reducing the total from 621 to 189 features that now could be annotated with a metabolite name (Figure 5B). We then selected metabolites that were significantly altered in either the 2-week or 4-week groups compared to controls, further narrowing down the list to 30 metabolites. Finally, we focused on metabolites that were significantly (i.e., adjusted *p*-value < 0.005) increased or decreased in both the 2-week and 4-week groups compared to controls to find the most robust markers. This stringent filtering yielded five candidate metabolites strongly associated with kidney injury in both the 2-week and 4-week groups (Figure 5C,D). The five candidates were indoxyl sulfate (IxS), pipecolic acid (Pip), galactonic acid (Gal), N-acetylneuraminic acid (Neu5Ac), and 5-sulfosalicylic acid (5-SSA; Figure 5E). To further confirm the identity of these metabolites and estimate their plasma concentrations, we conducted a targeted metabolomics analysis. This confirmed that all five metabolites were significantly increased in both the 2-week and 4-week groups compared to controls (Supplemental Figure S4A–E). Taken together, we identified IxS, Pip, Gal, Neu5Ac, and 5-SSA as plasma metabolites strongly and consistently associated with renal injury in both adenine-fed groups.

### 2.5. Validation of Candidate Metabolites in Male and Female Patients with CKD

**Figure 6:**
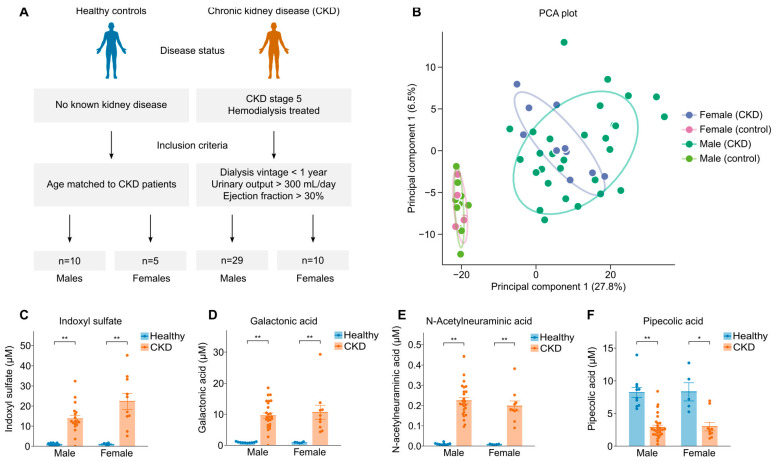
Plasma metabolomics of human CKD patients to validate candidate metabolites. (**A**) Schematic overview of selection criteria of the human cohort characteristics. (**B**) Principal component analysis (PCA) of the plasma metabolite profiles across patient groups. Principal component 1 explained 27.8% and principal component 2 explained 6.5% of the total variance in the dataset; each point represents an individual; and ellipses indicate 95% confidence intervals for each group. (**C**–**F**) Quantification of candidate metabolites by targeted metabolomics in plasma from CKD patients Injected concentration ranges of the clean compound were used to determine exact *m*/*z* values and retention times for each compound, which were then used to quantify plasma concentrations of (**C**) indoxyl sulfate (CKD men, *n* = 22; CKD women; *n* = 10, healthy men; *n* = 10, healthy women; *n* = 5), (**D**) galactonic acid (**E**) pipecolic acid (**F**), and N-acetylneuraminic acid. (**D**–**F**) CKD men, *n* = 29; CKD women, *n* = 10; healthy men, *n* = 10; healthy women, *n* = 5. Data are presented as mean ± SEM, and statistical significance was assessed using an unpaired Student’s *t*-test; *: *p* < 0.05; **: *p* < 0.001.

To assess the translatability of our findings, we repeated our plasma metabolomics analyses in a cohort of male and female patients with advanced CKD and compared them with age- and sex-matched healthy controls (Figure 6A). Key patient characteristics, biochemical parameters, dialysis status, and comorbidities are summarized in Supplemental Table S1. To get an understanding of the overall metabolic changes in the plasma of these patients, we first compared their plasma metabolomes using PCA. This analysis revealed a clear separation between the patients with advanced CKD and the healthy controls (Figure 6B), confirming that a distinct metabolic profile is associated with advanced CKD. Notably, no separation was observed between males and females within either the CKD or control group. To confirm that our five candidates were also relevant in human CKD, we quantified them in a targeted metabolomics analysis. We found that IxS, Gal, and Neu5Ac were significantly increased in the plasma of both male and female patients with advanced CKD compared to healthy controls (Figure 6C–E). In contrast to our findings in adenine-fed mice, we found that Pip was significantly decreased in both males and females with advanced CKD (Figure 6F). Moreover, 5-SSA could not be reliably quantified in the human samples due to retention time drift and changes in peak shape in the MS spectra and was therefore excluded from further analysis. We also measured the same panel of UTs as in the adenine mice and found reduced levels of tryptophan, alongside elevated levels of kynurenine, kynurenic acid, hippuric acid, and *p*-cresol sulfate in both male and female patients with advanced CKD (Supplemental Figure S5A–D,G). In contrast, and consistent with findings observed in the adenine mice, indoleacetic acid and phenylacetic acid were not significantly increased in the CKD patients of either sex (Supplemental Figure S5E,F). In summary, we found that the plasma metabolome of the advanced CKD patients differed from the healthy controls, regardless of sex. Among the five metabolite markers of renal injury, IxS, Gal, and Neu5Ac were consistently elevated, while Pip was decreased in both male and female patients with advanced CKD.

### 2.6. Correlation Analyses Between Candidate Metabolites Show Mild Associations with Diabetes and Strong Associations with Kidney Disease

**Figure 7:**
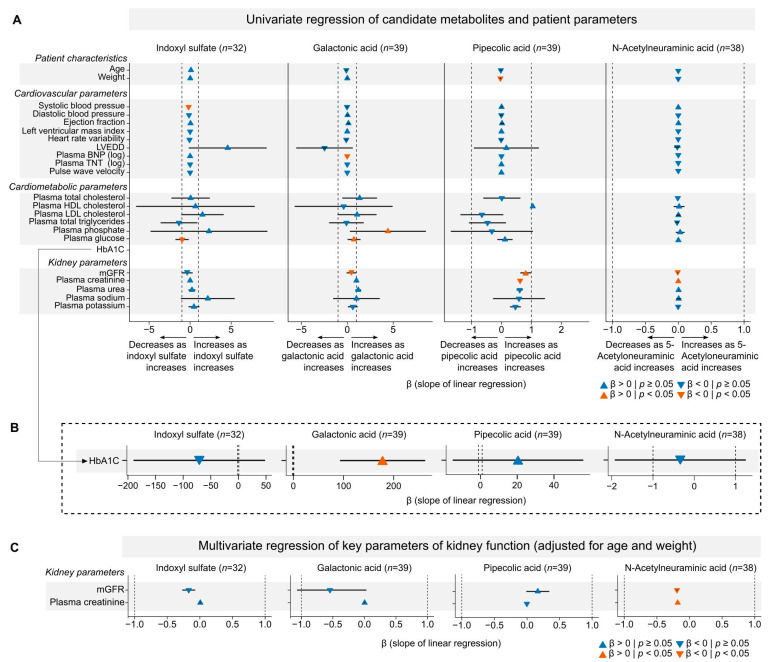
Associations between candidate metabolites and clinical parameters in CKD patients. (**A**,**B**) Forest plots of univariate linear regression analyses, evaluating associations between plasma concentrations of candidate metabolites and clinical parameters in the human CKD patients (male and female patients were combined). (**A**) Associations of candidate metabolites with demographic factors and cardiovascular, cardiometabolic, and kidney-related clinical parameters. Heart rate variability, or the N-N interval, is the time between two consecutive normal heartbeats in ms. (**B**) Associations of candidate metabolites with HbA1c (marker of glycemic control; the HbA1c correlations were placed on a separate *x*-axis, because the effect size was generally much higher than the remaining parameters). (**C**) Multivariate linear regression models of candidate metabolites with pre-dialytic plasma creatinine and measured glomerular filtration rate (mGFR), adjusted for age and body weight. (**A**–**C**) Triangles indicate direction and significance of associations, where blue triangles represent non-significant associations (*p* ≥ 0.05) and orange triangles represent significant associations (*p* < 0.05); upward- and downward-pointing symbols for positive and negative linear regression slopes, respectively; beta-values and 95% confidence intervals are shown for each regression.

The accumulation of small molecules, including UTs, in the plasma during CKD has been associated with an increased risk of developing secondary diseases such as cardiovascular disease (CVD) and diabetes [28,29]. To explore potential associations between the four potential biomarkers and these comorbidities, we performed multiple univariate regression analyses comparing the metabolite levels with established markers of CVD and diabetes. As shown in Figure 6, the metabolomics data did not show any sex differences; therefore, males and females were pooled to increase the statistical power of subsequent analyses. We first examined cardiovascular-related outcomes but found no strong associations between any of the metabolites and adverse CVD parameters, including cholesterol and triglyceride levels. Next, we investigated potential associations between the candidate metabolites and the diabetes-related markers, specifically glucose and HbA1c (a marker of long-term hyperglycemia). We found that Gal levels were significantly correlated with both plasma glucose and HbA1c (Figure 7A,B; Supplemental Figure S6B–E). The correlation with HbA1c remained significant after adjusting for age and body weight (Supplemental Figure S6A). Consistent with this finding, individuals with the highest Gal levels showed a markedly increased risk of having diabetes (odds ratio = 5.2, 95% CI: 1.2–29.6; Supplemental Figure S6F). Gal levels also tended to be higher in patients with diabetes compared to those without (Supplemental Figure S6G). However, these findings should be interpreted with caution given the limited sample size and wide confidence interval but warrant validation in larger independent cohorts. To expand our understanding of the associations between the four candidate metabolites (IxS, Gal, Pip, and Neu5Ac) and kidney disease, we performed a correlation analysis between their levels and measured GFR (mGFR), pre-dialytic plasma creatinine, and BUN in each patient (Figure 7A). We found that Gal, Pip, and Neu5Ac were all significantly associated with mGFR. In addition, Pip and Neu5Ac were also significantly correlated with pre-dialytic plasma creatinine levels. After adjusting for age and body weight, only Neu5Ac retained the significant association with mGFR and plasma creatinine (Figure 7C). IxS did not show significant correlations with either mGFR or creatinine. In summary, Gal showed a novel association with diabetes-related markers and an increased risk of diabetes. Additionally, Gal, Pip, and Neu5Ac were significantly associated with mGFR.

### 2.7. The Addition of Pipecolic Acid, Galactonic Acid and N-Acetylneuraminic Acid Improves Creatinine-Based GFR Predictions

**Figure 8:**
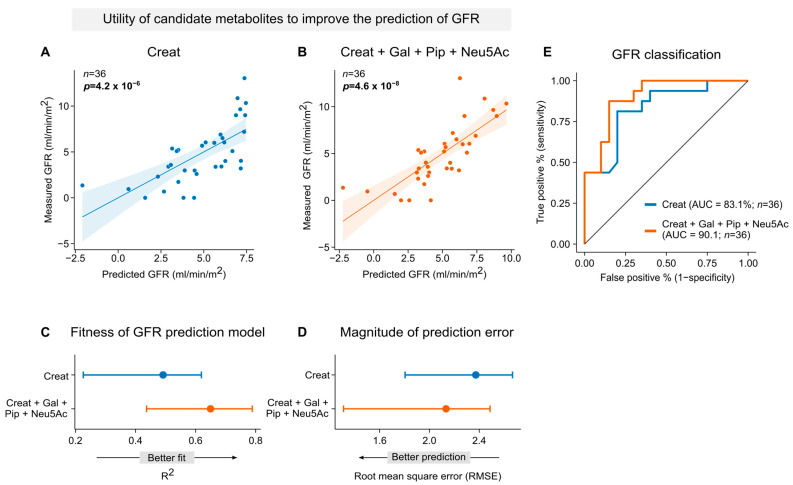
Combined candidate metabolite model improves GFR prediction compared with creatinine alone. (**A**,**B**) Scatter plots comparing measured GFR to GFR values predicted using linear regression models trained using 10-fold cross-validation. (**A**) Prediction model using pre-dialytic plasma creatinine (Creat) alone. (**B**) Combined prediction model including pre-dialytic creatinine (Creat), galactonic acid (Gal), pipecolic acid (Pip), and N-acetylneuraminic acid (Neu5Ac). (**C**) Forest plot of model fit as assessed by coefficient of determination (R^2^), comparing the creatinine-only model (blue) and the combined model (orange). R^2^ values with 95% confidence intervals are shown; higher values indicate better model fit. (**D**) Forest plot showing prediction error assessed by root mean square error (RMSE) for each model (blue: creatinine alone; orange: combined model). Lower RMSE values indicate better predictive accuracy. (**D**,**E**) Model uncertainty was assessed using bootstrap resampling (100 iterations), in which the dataset was repeatedly resampled with replacement and model performance (R^2^ and RMSE) recalculated to derive 95% confidence intervals. (**E**) Receiver operating characteristic (ROC) curves evaluating model performance for classifying individuals with GFR below the median. The combined model (orange) achieved an AUC of 90.1%, while the creatinine-only model (blue) achieved an AUC of 83.1%. The black diagonal line shows the performance of a non-informative classifier, serving as a reference for random chance.

We next assessed whether incorporating Gal, Pip, and Neu5Ac into pre-dialytic plasma creatinine-based models could enhance eGFR estimation in patients with advanced CKD. To assess predictive performance, we trained two linear regression models for GFR: one using creatinine alone and another using a combination of creatinine, Gal, Pip, and Neu5Ac. Both models produced GFR estimates that correlated significantly with mGFR (Figure 8A,B). However, the combination model outperformed the creatinine-only model, with an improved model fit (R^2^ = 0.62, 95% CI: 0.44–0.79 for the combination model versus R^2^ = 0.45, 95% CI: 0.23–0.62 for the creatinine model; Figure 8C) and a reduced root mean square error (RMSE; RMSE = 2.13 mL/min/m^2^, 95% CI: 1.31–2.49 for the combination model versus RMSE = 2.37 mL/min/m^2^, 95% CI: 1.80–2.67 for the creatinine model; Figure 8D). Furthermore, we evaluated the added predictive value of these metabolites for identifying individuals with low GFR. To do this, we trained a logistic regression model to estimate the probability of having a GFR below the median value of the cohort (i.e., 4.01 mL/min/m^2^), using either creatinine alone or a combination of creatinine with Gal, Pip, and Neu5Ac. The combination model achieved a higher prediction accuracy, with an AUC of 90.1%, compared to 83.1% for the creatinine-only model. In summary, by including Gal, Pip, and Neu5Ac in the pre-dialytic creatinine-based estimation of GFR, we could significantly improve both the continuous prediction of GFR and the classification of patients with low GFR. These findings are a proof-of-concept, showing that a multi-marker approach may enhance kidney function estimation in CKD patients beyond creatinine alone. Yet, validation in a larger cohort is needed.

## 3. Discussion

Although plasma creatinine remains a cornerstone of clinical kidney function assessment, it is influenced by non-renal factors, such as age, muscle mass, and diet [7,8]. Consequently, it is not an ideal standalone biomarker for the early detection or accurate monitoring of kidney disease. Growing evidence indicates that markers reflecting tubulointerstitial injury, rather than glomerular damage, may provide stronger predictive value for renal function decline [13]. To address this, we applied untargeted plasma metabolomics in an adenine-induced mouse model to identify candidate biomarkers, which we subsequently validated in patients with advanced CKD to assess their translational relevance.

Our study shows that galactonic acid (Gal), pipecolic acid (Pip), and N-acetylneuraminic acid (Neu5Ac) are promising candidate biomarkers of kidney injury. When combined with creatinine in a biomarker panel, these metabolites significantly improved pre-dialytic creatinine-based GFR estimation compared with creatinine alone. Together, these findings support the potential of a metabolite-based biomarker panel to strengthen the clinical evaluation of kidney function.

In this study, we found an association between galactonic acid (Gal) and elevated plasma glucose, impaired glycemic control, and increased prevalence of diabetes in CKD patients. Elevated plasma Gal has previously been linked to gut dysbiosis [30] and diabetes [31], both common comorbidities in CKD. In addition, previous studies in humans [32] and rodent models of diabetic nephropathy [33] have reported that higher Gal levels are associated with progression to end-stage kidney disease. Collectively, these observations suggest that Gal may serve as a biomarker for CKD, particularly in patients with diabetes. However, validation in a broader CKD cohort is needed, as well as mechanistic studies.

Our data showed that pipecolic acid (Pip) increased in the plasma of adenine-fed mice but was significantly reduced in the CKD patients. This species-specific discrepancy is consistent with previous studies, which found elevated Pip in mouse models of kidney disease [34,35] but decreased levels in humans with CKD [32,36]. Pip is derived from the degradation of lysine, a process that occurs mainly in the liver and brain [37]. Rinschen et al. demonstrated accelerated lysine degradation in a mouse model of hypertensive nephropathy [38], which may explain the increased Pip levels observed in our mice. These species-specific differences likely reflect fundamental variations in lysine metabolism between humans and mice, underscoring the need for a translational approach in biomarker discovery.

In our study, N-acetylneuraminic acid (Neu5Ac) showed the strongest correlations with both mGFR and plasma creatinine, in line with previous studies [39,40,41]. Neu5Ac is a key constituent of the glomerular filtration barrier [42], and elevated plasma levels have been reported in patients with glomerular kidney injury [43]. Despite this, Neu5Ac is mainly studied in the context of cardiovascular disease, where it has been implicated in the pathogenesis of heart failure [44] and myocardial infarction [45]. Our findings suggest that Neu5Ac may serve as a biomarker for CKD, and possibly for identifying CKD patients at high risk of cardiovascular complications; however, this hypothesis requires further study.

Our study shows that the adenine mouse model is well-suited for identifying translational biomarkers of human CKD. In addition to the identified biomarkers, we observed increased levels of several tryptophan-derived metabolites in adenine-fed mice, including kynurenine, xanthurenic acid, kynurenic acid, and IxS, all of which have been previously reported as potential clinical biomarkers in large CKD cohorts [46,47,48,49]. We also detected elevated levels of pseudouridine, C-mannosyltryptophan, acetylcarnitine, and tiglylcarnitine, metabolites that have each been linked to kidney dysfunction [14,46,50].

Our metabolic profiling of adenine-fed mice revealed an accumulation of organic acids and benzenoids, accompanied by a reduction in fatty acids. These findings are consistent with previous studies suggesting that the elevated organic acid levels in CKD reflect a metabolic shift in the kidneys, characterized by the accumulation of amino acid catabolites and intermediates of the TCA cycle, such as fumarate and citrate [51,52]. Together with altered energy metabolism, we also observed elevated levels of benzenoids such as IxS and *p*-cresol sulfate, which are well-known gut-derived UTs [53]. This increase in the metabolites likely reflects impaired renal clearance and gut dysbiosis, both commonly observed in human CKD [54,55].

In addition to identifying plasma biomarkers, we observed a reduced expression profile of proximal tubule transporters (OAT1, OAT3, and OCT2) at both mRNA and protein levels in adenine-fed mice and CKD patients, consistent with previous studies [56,57,58]. These transporters facilitate the urinary excretion of a wide range of small metabolites and may contribute to the accumulation of Gal, Pip, and Neu5Ac. Supporting this hypothesis, increased plasma Gal has been reported in OAT3 KO mice [59,60] with levels approximately 1.5-fold higher than in wild-type mice. However, our adenine-fed mice exhibited a 5–6-fold increase in Gal, indicating that reduced excretion alone cannot account for the observed rise and that altered Gal metabolism also contributes. Similarly, the observed changes in Pip levels are unlikely to be explained by reduced transporter activity, as Pip levels were decreased in CKD patients. Moreover, prior studies using OAT1 and OAT3 KO mice did not report increased Neu5Ac [54,59,61], suggesting that Neu5Ac accumulation may arise directly from kidney injury rather than impaired excretion. Taken together, these findings suggest that the increases in Pip and Neu5Ac primarily reflect kidney injury, whereas elevated Gal levels likely result from a combination of reduced transporter activity and altered metabolism.

A key strength of this study is the use of the adenine mouse model to first identify plasma metabolites associated with renal injury, followed by validation in a patient cohort. This approach minimizes the risk of confounding by non-renal factors and ensures that the identified biomarkers accurately reflect renal injury. In addition, we used the Quadrupole Exactive Plus Orbitrap MS for the untargeted analysis, offering superior resolution and mass accuracy compared with more commonly used platforms [62]. Finally, we applied stringent feature-filtering criteria to the dataset, followed by targeted validation using internal standards, to accommodate the well-known challenges of metabolite annotation [63].

A limitation of this study is the fact that we only included patients with advanced CKD undergoing hemodialysis (HD), which restricted our prediction model to GFR values between 0 and 14 mL/min/m^2^, whereas in the broader CKD population (stage 3–5), GFR can be as high as 59 mL/min/m^2^. Moreover, because creatinine levels decrease during dialysis, plasma creatinine is a challenging functional measure of kidney function in HD patients. However, previous studies have shown that plasma creatinine rebounds to near pre-dialysis levels within hours after dialysis treatment [64]. Therefore, to minimize the influence of this immediate post-dialysis drop on our prediction analyses, plasma samples in this study were collected immediately before dialysis. Future studies should evaluate whether our prediction model can detect GFR changes in patients with CKD stage 1–4. In addition, our model would greatly benefit from external validation using independent data sets. Another limitation is the small sample size of our human cohort, which may have prevented detection of certain correlations between metabolite levels and clinical parameters. For instance, IxS could be quantified in only 32 of the 39 patients and showed no association with either reduced mGFR or any CVD parameters, in contrast to previous studies [49,65]. In addition, the inclusion of locally recruited healthy volunteers as controls may introduce selection bias; however, age- and sex-matching was performed to enhance comparability with the CKD cohort.

## 4. Conclusions

In conclusion, we identified galactonic acid (Gal), pipecolic acid (Pip), and N-acetylneuraminic acid (Neu5Ac) as promising candidate biomarkers of renal injury. In our hands, combining these metabolites into a biomarker panel with creatinine substantially improved GFR estimation compared with creatinine alone. This biomarker panel could potentially be used to improve clinical monitoring of kidney function. However, validation studies in larger cohorts are needed, as well as studies assessing longitudinal changes in Gal, Pip, and Neu5Ac levels in CKD patients, the effects of dialysis on their plasma levels, and whether these metabolites are associated with clinical outcomes.

## 5. Materials and Methods

### 5.1. Human CKD Patients and Healthy Controls

Stored human plasma samples, kept at −80 °C, were obtained from the SAFIR cohort. SAFIR (acronym for SAving residual renal Function in hemodialysis patients receiving IRbesartan) was a randomized double-blinded placebo-controlled, multicenter trial (Clinical Trials ID: NCT00791830) [66,67]. Inclusion began in May 2009, and the last patient’s last visit was in December 2012. Patients were recruited from six hospitals in Denmark. All sites were monitored by a local independent Good Clinical Practice unit. The samples used in the current study were collected prior to HD treatment at baseline before intervention with either Irbesartan or placebo. Details regarding blood sampling as well as inclusion and exclusion criteria have previously been published [66,67,68]. Briefly, inclusion criteria were dialysis vintage ≤ 1 year, urinary output > 300 mL/24 h, left ventricular ejection fraction > 30%, pre-dialytic systolic blood pressure > 110 mmHg prior to admission, and without any episodes of myocardial infarction or unstable angina pectoris in the last three months prior to admission. The GFR was measured by Cr-EDTA clearance [68]. To assess metabolite stability following long-term storage, plasma samples from two patients currently undergoing chronic HD treatment at the Department of Renal Medicine, Aarhus University Hospital, were included as analytical controls and compared with the SAFIR cohort. To obtain age- and sex-matched non-CKD controls, fifteen healthy volunteers were recruited locally. As these participants were enrolled through convenience sampling, the potential for selection bias cannot be excluded.

### 5.2. Adenine-Induced Kidney Disease

Animal experiments were performed using a total of 27 eight-week-old male C57BL/6NRj mice (Janvier Labs, Le Genest Saint Isle, France). The mice were housed in groups of four under controlled environmental conditions: a 12 h:12 h light-dark cycle, constant temperature of 21  ±  2 °C, and humidity of 55 ± 2%. The mice had free access to both food and water. The mice were allowed to acclimatize for one week prior to the start of the experiment. Mice received either a casein-based diet (control group; *n* = 11) or the same diet supplemented with 0.2% adenine to induce kidney injury for 2 weeks (2-week group; *n* = 8) or 4 weeks (4-week group; *n* = 8). To make sure that kidney function did not change over time, control mice were euthanized along with the adenine-fed mice at the respective time point. Body mass, food, and water intake were recorded daily until day 16, and every other day thereafter. At sacrifice, the mice were anesthetized with 5% sevoflurane and placed on a heating pad. Blood was collected through cardiac puncture into heparin-coated tubes. Both kidneys were excised and weighed; the left kidney was dissected into cortex and inner medulla for RNA and protein extraction, while the right kidney was fixed in 4% formaldehyde for histology. Data from the control and 4-week groups describing phenotypic changes in the kidneys (Figure 1B–G, Figure 2A–C, Supplemental Figures S1–S3) have been published previously [23] and are included here as reference data. The 2-week group represents newly generated and previously unpublished data. All samples originate from the same animal experiment, and all groups were processed in parallel under identical conditions to ensure comparability. To avoid reanalysis bias, no statistical comparisons were performed on the previously published data in isolation; instead, all analyses were conducted across groups within the current dataset.

### 5.3. Plasma and Urine Biochemistry

Plasma creatinine levels were measured using a Creatinine Assay Kit (Sigma Aldrich, St. Louis, MI, USA). Blood urea nitrogen (BUN) levels were measured using a Urea Nitrogen Colorimetric Detection Kit (Thermo Fisher Scientific, Waltham, MA, USA).

### 5.4. Quantitative PCR

Kidney cortex tissue was homogenized, and the RNA was purified using a NucleoSpin^®^ RNA kit (Macherey Nagel, Düren, Germany), as described previously [23]. cDNA was synthesized from 1 µg of total RNA using the RevertAid First Strand synthesis kit (#K1622, Thermo Fisher Scientific, Waltham, MA, USA), and qPCR was performed with a Maxima SYBR Green qPCR Master Mix (Thermo Fisher Scientific, Waltham, MA, USA). Gene expression was calculated using the ΔCt method with *Gapdh* as the reference gene. Primer sequences are listed in Supplemental Table S2.

### 5.5. Western Blotting

Kidney cortex tissue was homogenized and separated by SDS-PAGE, followed by transfer to a nitrocellulose membrane, as described previously [23]. Total protein was visualized using the No-Stain Protein Labeling Reagent (#A44717, Thermo Fisher Scientific, Waltham, MA, USA), following the manufacturer’s protocol. The membrane was blocked in skim milk for 1 h, incubated overnight at 4 °C with the primary antibodies (Supplemental Table S3), and then incubated with horseradish peroxidase (HRP)-conjugated secondary antibody (Supplemental Table S3). The band intensities were normalized to total protein staining.

### 5.6. Immunohistochemistry and Histology

The complete immunohistochemistry protocol has been described previously [23]. Briefly, the right kidney was removed and immersed in 4% paraformaldehyde for 1 h, rinsed in PBS, dehydrated in a series of alcohol, and embedded in paraffin. Tissue sections (2 µm) were deparaffinized, rehydrated, rinsed, and subjected to antigen retrieval using Tris-EDTA buffer (except for the OCT2 staining, where citrate buffer was used). Sections were blocked and incubated overnight at 4 °C with primary antibodies (Supplemental Table S3). The next day, the sections were incubated with HRP-conjugated secondary antibodies (Supplemental Table S3) for 1 h, followed by 10 min of DAB staining. Imaging was performed on a Nikon Ti2 Eclipse inverted microscope (20× air objective; numerical aperture [NA] = 0.45) with automatic stitching. DAB signals were extracted using the Color Deconvolution plugin in FIJI. In addition, 3 µm-thick kidney sections were stained with H&E. The rehydrated and rinsed sections were stained with Mayer’s hematoxylin for 5 min, rinsed, and counterstained with Eosin Yellow for 30 s before being dehydrated and mounted with a coverslip. Imaging was performed using an Olympus VS120 Virtual Slide Scanner (40× air objective; NA = 0.95). Image stitching was performed automatically.

### 5.7. Immunofluorescence and Image Analysis

Immunofluorescence (IF) staining of KIM1 and AQP1 was done in a multiplex fashion using coverslip-mounted paraffin-embedded tissue sections, as described in detail previously [23,69]. Briefly, kidney sections (2 µm) were placed on gelatine-coated round coverslips. The sections were deparaffinized, rehydrated, rinsed, subjected to antigen retrieval, and incubated with primary antibodies overnight (Supplemental Table S3). The following day, the coverslips were rinsed in PBS and incubated with secondary antibodies (Supplemental Table S3) for 1 h in the dark. After rinsing in PBS, the coverslips were placed in an imaging chamber (Live Cell Instrument Co., Namyangju-si, Republic of Korea) and imaged using a Nikon Eclipse Ti2 microscope (60× oil objective, NA = 1.45). After imaging, the sections were stripped and re-incubated with the second round of primary antibodies. The KIM1 antibodies (R&D Systems, Minneapolis, MN, USA) were used in the first round and the AQP1 antibodies (Sigma-Aldrich Corporation, St. Louis, MO, USA) were used in the second round. IF staining of αSMA and PDGFRβ was performed using tissue sections mounted on microscope slides, as described in detail previously [23]. Here, the kidney sections were attached to SuperFrost+ microscope slides (Hounisen, Skanderborg, Denmark) instead of coverslips. Otherwise, the protocol is identical to the one described above. The images were acquired using an Olympus VS120 Virtual Slide Scanner (40× air objective; NA = 0.95).

The number of KIM1-positive tubules was counted in the cortical area. First, the cortical area was manually selected. Thereafter, a threshold-based mask was created to segment positive from negative tubules. The threshold was based on the average fluorescence intensity of positive tubules. The number of positively stained tubules was then automatically quantified using FIJI (“Analyze” > “Analyze Particles”).

### 5.8. Plasma Deproteination and External Standards

Plasma samples (50 µL) from mice and humans were incubated with 200 µL of −20 °C liquid chromatography–mass spectrometry (LC-MS) Grade Methanol (LC-MS Grade, 047192-K2, Thermo Fisher Scientific, Waltham, MA, USA) in Low Protein Binding Microcentrifuge Tubes (#90410, Thermo Fisher Scientific, Waltham, MA, USA), vortexed at 1400 RPM for 5 min at 5 °C (Eppendorf ThermoMixer, VWR, Blanchardstown, Ireland), and incubated overnight at −20 °C. The following day, samples were vortexed (3000 RPM for 5 s) and centrifuged (15,000× *g*, 20 min, 5 °C), and 200 µL of supernatant was collected. From this, 20 µL of each sample was pooled in a quality control (QC) sample. Samples were dried using a vacuum concentrator (SpeedVac, VWR, Blanchardstown, Ireland) and reconstituted in 36 µL LC-MS buffer (0.2% formic acid in water), vortexed (3000 RPM for 5 s), sonicated to remove air, and stored at −20 °C until use. Standards were either dissolved in DMSO (e.g., kynurenic acid, hippuric acid, indoxyl sulfate) and diluted in LC-MS-grade water or directly dissolved in LC-MS-grade water (i.e., Pip, Neu5Ac) and stored at −20 °C until use. Working solutions were prepared across a range reflecting expected physiological concentrations (Supplemental Table S4).

### 5.9. Targeted and Untargeted Metabolomics

Deproteinated human and mouse plasma samples (7–10 µL) were analyzed by LC-MS/MS on a Vanquish Horizon LC system coupled with a Q Exactive Plus Orbitrap MS (Thermo Fisher Scientific) using an Accucore Biphenyl column (Thermo Fisher Scientific, Waltham, MA, USA), as previously described [70]. Analyses were performed in both positive and negative ionization modes using previously described settings [70]. MS data were acquired from the samples, and the identification of the features was performed using Compound Discover (version [v] 3.3, Thermo Fisher Scientific; Supplemental Table S5). Feature identification was performed on the QC samples using MS/MS data. Spectral matching was conducted against the mzCloud database to identify and annotate metabolites with higher certainty. Further information regarding the methodological workflow of the untargeted analysis can be found in Supplemental Table S5. Subsequent data analysis and visualization were performed using R (v 4.4.1) and RStudio (v 2024.04.2 + 764). Standardized metabolite names and mapping of metabolite classes were performed using RefMet [71] and supported by manual look-up in a KEGG database. For the targeted analysis of the standards, the serial dilutions were injected into the LC-MS and run on either positive or negative ionization mode. The peak integration of parent masses ([M + H]^+^ or [M-H]^−^) was performed with Skyline (v 24.1.0.199) [72]. The slopes of the calibration curves were generated on log10-transformed peak areas, and the concentration of the standards was determined by linear regression analysis.

### 5.10. Statistics

Data are presented as mean ± SEM unless stated otherwise. Normality was assessed using the Shapiro–Wilk test. Group comparisons in the animal study were performed using one-way ANOVA with Tukey’s post hoc test. When the assumption of normality was not met, non-parametric tests (Kruskal–Wallis) were used. Comparisons of plasma metabolite levels between healthy controls and CKD patients were performed using an unpaired Student’s *t*-test. Statistical analyses and visualizations were conducted in R (v 4.4.1) using RStudio (v 2024.04.2 + 764) and the Tidyplots package [73]. A *p*-value < 0.05 was considered statistically significant. Pathway analysis was performed using the Pathway Analysis tool in Metaboanalyst 6.0; here, a table containing peak areas for all features within each sample was uploaded, and the data was normalized using “Normalized by sum”, transformed using “Log transformation (base 10)”, and scaled using “Mean centering”. The KEGG pathway from *Homo Sapiens* was used as a reference database to support the assignment of metabolite common names.

Univariate and multivariate regression analyses were performed in RStudio using the function lm() from the base stats package. Odds ratios were calculated using the function oddsratio() from the epitools package (v. 0.5-10.1). Logistic regression models were built, as proof-of-concept, to classify individuals as having below- or above-median (4.01 mL/min/m^2^) mGFR based on selected metabolite predictors. For each candidate prediction model, the dataset was randomly partitioned into 10 folds, with iterative training on nine folds and validation on the remaining fold (10-fold cross-validation) using the caret() package in R. To assess uncertainty in model performance, a bootstrap resampling approach (100 iterations) was applied. In each iteration, the dataset was resampled with replacement, the model was refitted, and performance metrics (R^2^ and RMSE) were recalculated. Predicted probabilities for the “below-median” class were extracted and used to compute receiver operating characteristic curves with the pROC package (v. 1.19.0.1).

## Data Availability

The original contributions presented in this study are included in the article/Supplementary Material. Further inquiries can be directed to the corresponding author(s).

## Supplementary Materials

The following supporting information can be downloaded at: https://www.mdpi.com/article/10.3390/toxins18050225/s1, Supplemental Figure S1. Histology and proximal tubule health. (A) H&E staining of kidney sections in the control, 2-week, and 4-week groups (top panels). Inserts show magnified regions (bottom panels). Areas of fibrosis and tubulointerstitial injury are encircled with a dashed line; representative images are shown (n = 5); top scale bar = 1 mm; bottom scale bar = 50 µm. Images were acquired on an Olympus VS120 Virtual Slide Scanner (40× air objective). (B) Immunofluorescence staining for Aquaporin 1 (AQP1) in kidney sections mounted on coverslips from controls, highlighting healthy proximal tubules. Insert shows the magnified region (white rectangle); representative images are shown (n = 5); left scale bar = 1 mm; right scale bar = 100 µm. Images were acquired on a Nikon Eclipse Ti2 microscope (60× oil objective). (A,B) Images from the control and 4-week group have previously been published [23]; Supplemental Figure S2. α Smooth muscle actin and PDGFRβ co-staining. Immunofluorescence staining of kidney tissue sections for Hoechst (cyan, nuclei), α Smooth muscle actin (αSMA; green, marker of myofibroblasts) and PDGFRβ (magenta, marker of fibroblasts) in the control, 2-week, and 4-week groups. The images cover the outer cortex (top) and outer medulla (bottom); the stainings are shown individually and merged; inserts show magnified regions (white rectangles); white arrow heads point at overlapping αSMA and PDGFRβ staining, indicating activated myofibroblasts. Representative images are shown (n = 5); left scale bar = 100 µm; right scale bar = 40 µm. Images were acquired on an Olympus VS120 Virtual Slide Scanner (40× air objective). Images from the control and 4-week group have previously been published [23]; Supplemental Figure S3. Kidney cortex inflammation and fibrosis. (A–F) Kidney cortex mRNA expression levels of (A) Ccl2, (B) Tnf, (C) Il6, (D) Tgfb1, (E) Fn1, and (F) Col1a1 were assessed by qPCR, normalized to Gapdh expression, and expressed as fold change compared with control levels (2-week and 4-week groups n = 8; controls n = 10); data are presented as mean ± SEM and statistical significance was evaluated by either one-way ANOVA followed by Tukey–Kramer post hoc test or Kruskal–Wallis test; *: *p* < 0.05; **: *p* < 0.001. (A–F) Data from the control and 4-week group have previously been published [23]; Supplemental Figure S4. Targeted plasma metabolomics from adenine-fed mice. (A–L) Targeted quantification of plasma metabolites in control (n = 7), 2-week (n = 6), and 4-week (n = 7) groups of (A) Indoxyl sulfate, (B) Pipecolic acid, (C) Galactonic acid, (D) N-acetylneuraminic acid, (E) 5-Sulfosalicylic acid, (F) Kynurenic acid, (G) Hippuric acid, (H) Indoleacetic acid, (I) Kynurenine, (J) Tryptophan, (K) Phenylacetic acid, and (L) p-cresol sulfate. Data are presented as mean ± SEM and statistical significance was assessed using one-way ANOVA followed by Tukey–Kramer post hoc test or Kruskal–Wallis test; *: *p* < 0.05; **: *p* < 0.001; Supplemental Figure S5. Targeted plasma metabolomics from CKD patients. (A–G) Targeted quantification of plasma metabolites in human CKD patients (CKD men, n = 29; CKD women, n = 10,) and healthy controls (healthy men, n = 10; healthy women, n = 5). (A) Tryptophan, (B) Kynurenine, (C) Kynurenic acid, (D) Hippuric acid, (E) Indoleacetic acid, (F) Phenylacetic acid, and (G) p-cresol sulfate. Data are presented as mean ± SEM and statistical significance was assessed using one-way ANOVA followed by Tukey–Kramer post hoc test; *: *p* < 0.05; **: *p* < 0.001; Supplemental Figure S6. Galactonic acid and diabetes. (A) Multivariate linear regression analyses of galactonic acid against HbA1c and glucose adjusted for age and body weight. Triangles indicate direction and significance of associations: blue triangles represent non-significant associations (*p* ≥ 0.05), with upward- and downward-pointing symbols for positive and negative slopes, respectively; orange triangles represent significant associations (*p* < 0.05), again indicating directionality. β values and 95% confidence intervals are shown for each regression. (B,C) Linear regression analyses showing the relationship between plasma galactonic acid (B) plasma glucose and (C) plasma HbA1c. (D,E) plasma galactonic acid levels divided in tertiles from indicating low (n = 15), middle (n = 13) and high (n = 13) levels of galactonic acid and the corresponding plasma concentrations of (D) glucose and (E) HbA1c. (F) The odds ratio for having diabetes between patients with galactonic acid levels below median compared with those above. The odds ratio is shown with 95% confidence intervals. (G) Galactonic acid concentrations stratified by diabetes status, either not having (No, n = 24) or having diabetes (Yes, n = 13); Supplemental Table S1. Patient characteristics; Supplemental Table S2. Primer sequences used for Qpcr; Supplemental Table S3. Antibodies used for immunoblotting, immunohistochemistry, and immunofluorescence; Supplemental Table S4. Pure chemicals used as standards for targeted metabolomics; Supplemental Table S5. Pipeline of the statistical methodology for identification of features in Compound Discover.

## Abbreviations

5-SSA	5-sulfosalicylic acid
αSMA	alpha-smooth muscle actin
ANOVA	analysis of variance
AQP1	aquaporin 1
AUC	area under the curve
BCRP	breast cancer resistance protein
BUN	blood urea nitrogen
CI	confidence interval
CKD	chronic kidney disease
CVD	cardiovascular disease
eGFR	estimated glomerular filtration rate
Gal	galactonic acid
GC-MS	gas chromatography–mass spectrometry
GFR	glomerular filtration rate
HbA1c	hemoglobin A1c
HD	hemodialysis
HRP	horseradish peroxidase
IF	immunofluorescence
IxS	indoxyl sulfate
KIM1	Kidney injury molecule 1
KO	knockout
LC-MS/MS	liquid chromatography–tandem mass spectrometry
mGFR	measured glomerular filtration rate
MATE1	multidrug and toxin extrusion protein 1
MRP4	multidrug resistance–associated protein 4
Neu5Ac	N-acetylneuraminic acid
OAT1	organic anion transporter 1
OAT3	organic anion transporter 3
OATP4C1	organic anion transporting polypeptide 4C1
OCT2	organic cation transporter 2
PCA	principal component analysis
PDGFRβ	platelet-derived growth factor receptor beta
Pip	pipecolic acid
PT	proximal tubule
QC	quality control
qPCR	quantitative polymerase chain reaction
RMSE	root mean square error
ROC	receiver operating characteristic
SAFIR	SAving residual renal Function in hemodialysis patients receiving IRbesartan study
TCA cycle	tricarboxylic acid cycle
UTs	uremic toxins.
