# Supplementary material for: Plasma Metabolomics Reveals a Shared Metabolomic Profile in Experimental and Human Chronic Kidney Disease
Source: Toxins (Basel). 2026 May 9;18(5):225. doi: 10.3390/toxins18050225 (PMC13211520; doi:10.3390/toxins18050225)
Supplement: Supplementary file 1 [file toxins-18-00225-s001.zip › toxins-4240875-supplementary.pdf]

Supplementary Materials: Plasma Metabolomics Reveals a Shared Metabolomic Profile in Experimental and Human Chronic Kidney Disease

Søren H. Elsborg, Jasmine C. L. Atay, Johan Palmfeldt, Christian Daugaard Peters, Krista Dybtved Kjærgaard, Henricus A. M. Mutsaers and Rikke Nørregaard

Supplemental Figure S1. H&E and AQP1 staining.

Supplemental Figure S2. α Smooth muscle actin and PDGFRβ co-staining.

Supplemental Figure S3. Kidney cortex inflammation and fibrosis**.**

Supplemental Figure S4. Targeted plasma metabolomics from adenine-fed mice.

Supplemental Figure S5. Targeted plasma metabolomics from human CKD patients.

Supplemental Figure S6. Galactonic acid and diabetes.

Supplemental Table S1. Patient characteristics.

Supplemental Table S2. Primer sequences used for qPCR.

Supplemental Table S3. Antibodies used for immunoblotting, immunohistochemistry, and immunofluorescence.

Supplemental Table S4. Pure chemicals used as standards for the targeted metabolomics.

Supplemental Table S5. Pipeline of the statistical methodology for identification of features in Compound Discover.

Supplementary Figures

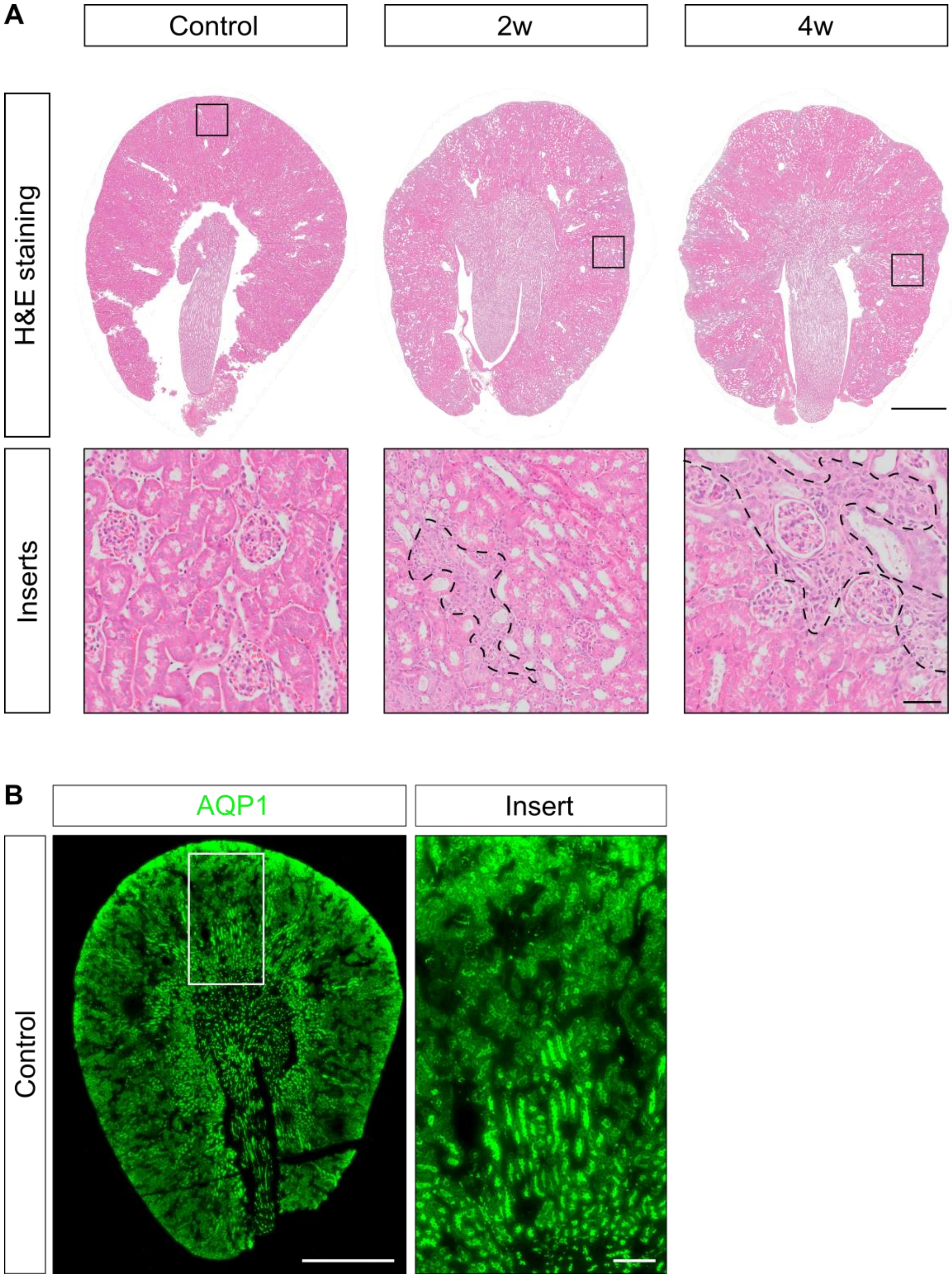

Supplemental Figure S1. Histology and proximal tubule health. (**A**) H&E staining of kidney sections in the control, 2-week, and 4-week groups (top panels). Inserts show magnified regions (bottom panels). Areas of fibrosis and tubulointerstitial injury are encircled with a dashed line; representative images are shown (*n*=5); top scale bar = 1 mm; bottom scale bar = 50 µm. Images were acquired on an Olympus VS120 Virtual Slide Scanner (40× air objective). (**B**) Immunofluorescence staining for Aquaporin 1 (AQP1) in kidney sections mounted on coverslips from controls, highlighting healthy proximal tubules. Insert shows the magnified region (white rectangle); representative images are shown (*n*=5); left scale bar = 1 mm; right scale bar = 100 µm. Images were acquired on a Nikon Eclipse Ti2 microscope (60× oil objective). (**A**-**B**) Images from the control and 4-week group have previously been published [23].

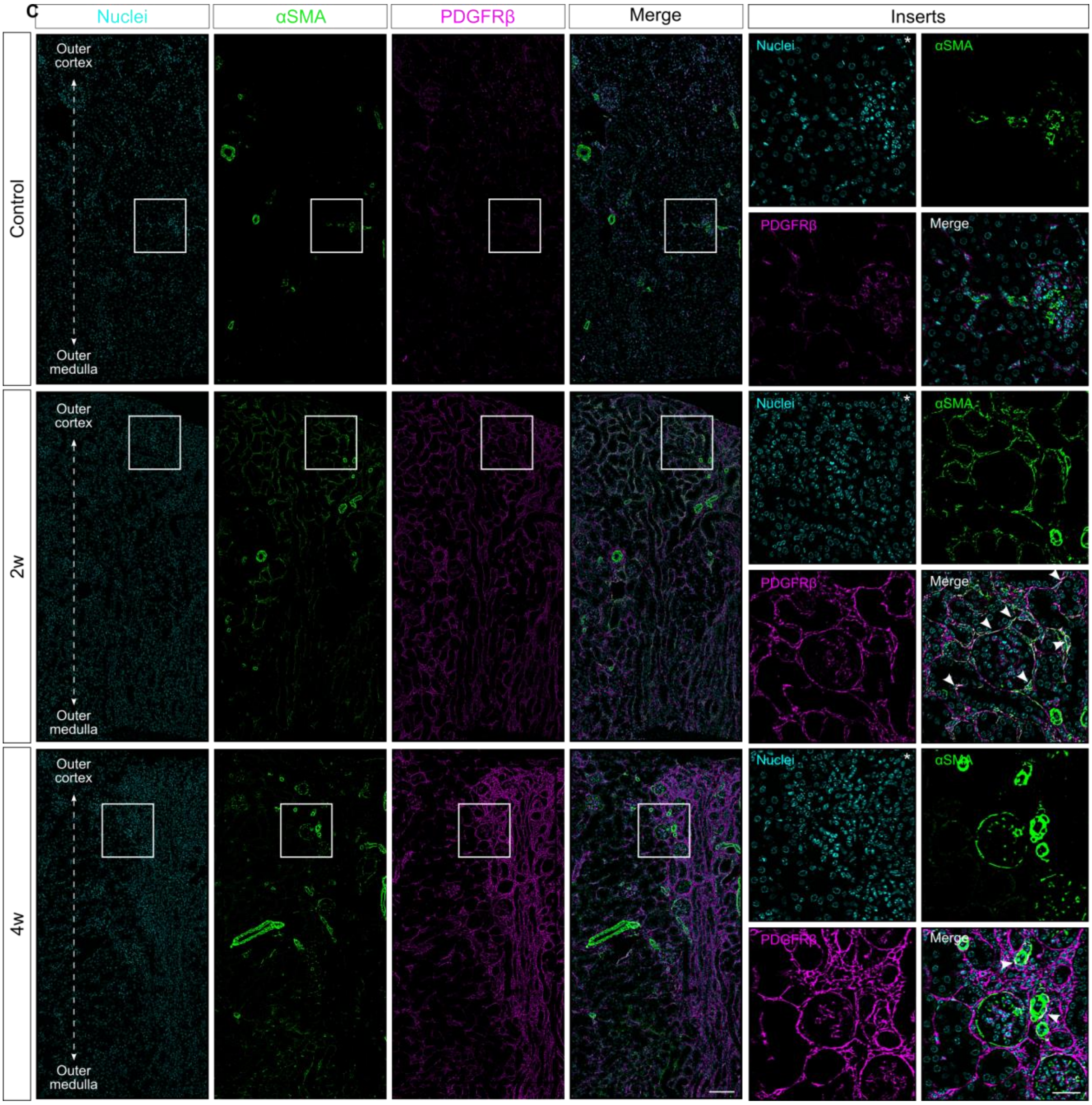

Supplemental Figure S2. α Smooth muscle actin and PDGFRβ co-staining. Immunofluorescence staining of kidney tissue sections for Hoechst (cyan, nuclei), α Smooth muscle actin (αSMA; green, marker of myofibroblasts) and PDGFRβ (magenta, marker of fibroblasts) in the control, 2-week, and 4-week groups. The images cover the outer cortex (top) and outer medulla (bottom); the stainings are shown individually and merged; inserts show magnified regions (white rectangles); white arrow heads point at overlapping αSMA and PDGFRβ staining, indicating activated myofibroblasts. Representative images are shown (n=5); left scale bar = 100 µm; right scale bar = 40 µm. Images were acquired on an Olympus VS120 Virtual Slide Scanner (40× air objective). Images from the control and 4-week group have previously been published [23].

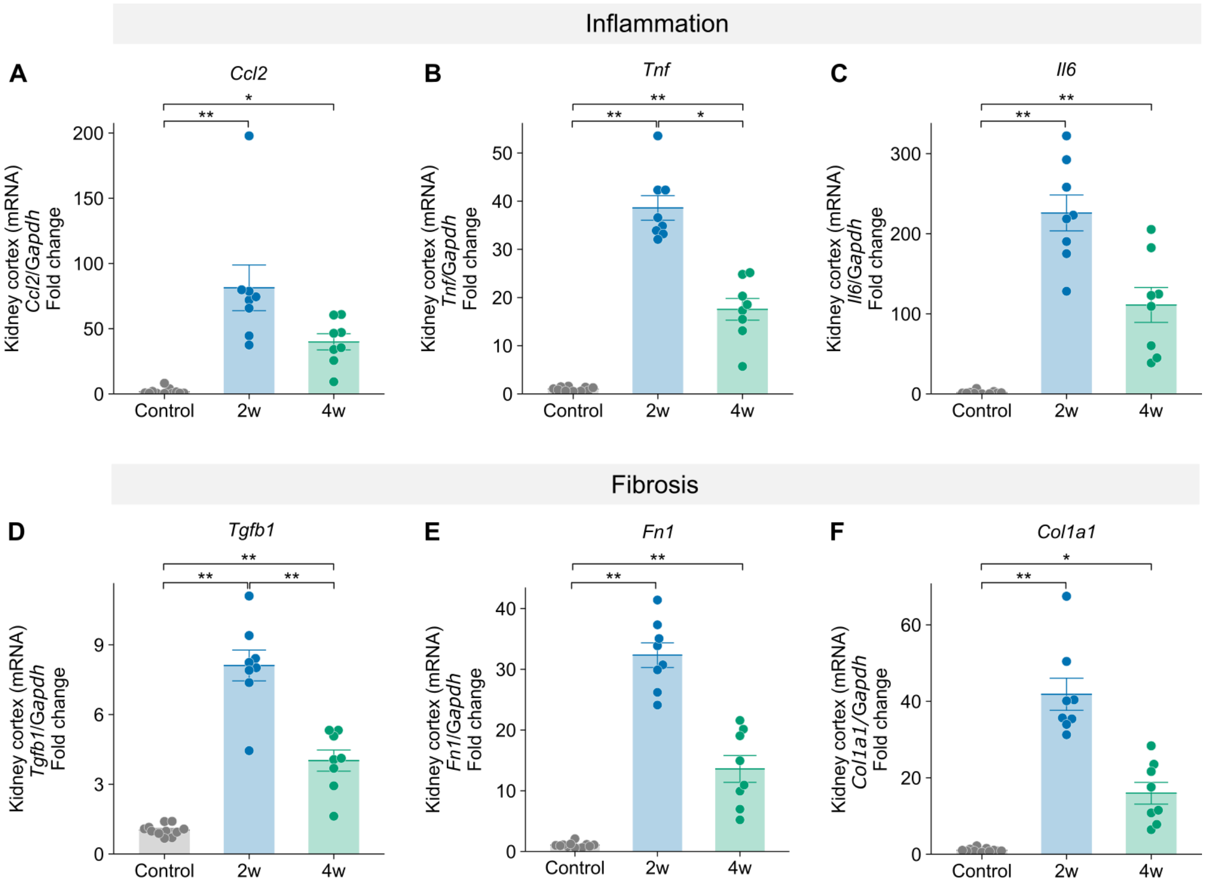

Supplemental Figure S3. Kidney cortex inflammation and fibrosis. (**A**-**F**) Kidney cortex mRNA expression levels of (**A**) *Ccl2*, (**B**) *Tnf*, (**C**) *Il6*, (**D**) *Tgfb1*, (**E**) *Fn1, and* (**F**) *Col1a1* were assessed by qPCR, normalized to *Gapdh* expression, and expressed as fold change compared with control levels (2-week and 4-week groups n=8; controls n=10); data are presented as mean ± SEM and statistical significance was evaluated by either one-way ANOVA followed by Tukey–Kramer post hoc test or Kruskal–Wallis test; *: $p < 0.05$; **: $p < 0.001$. (**A**-**F**) Data from the control and 4-week group have previously been published [23].

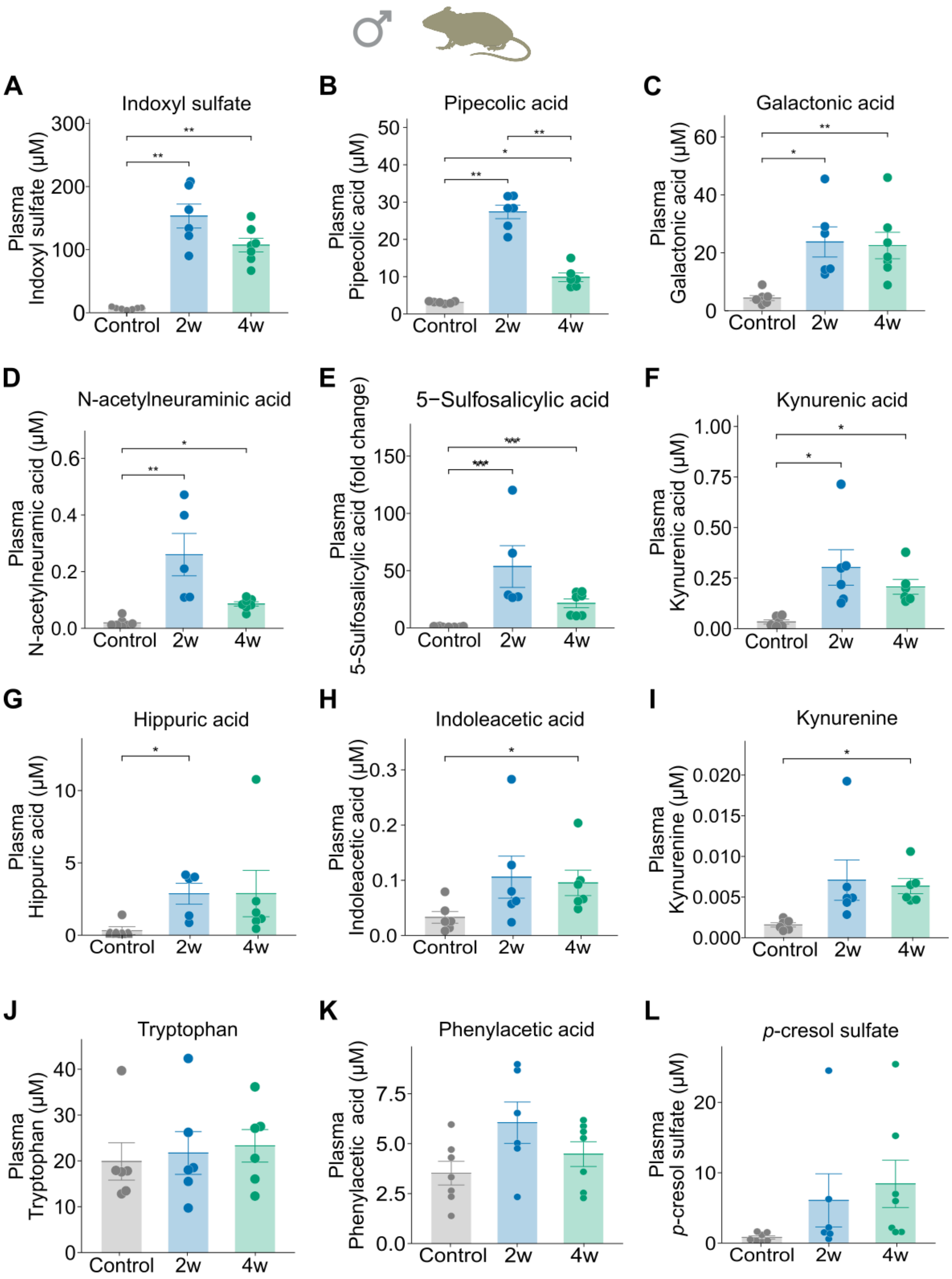

Supplemental Figure S4. Targeted plasma metabolomics from adenine-fed mice. (**A**-**L**) Targeted quantification of plasma metabolites in control (n=7), 2-week (n=6), and 4-week (n=7) groups of (**A**) Indoxyl sulfate, (**B**) Pipecolic acid, (**C**) Galactonic acid, (**D**) N-acetylneuraminic acid, (**E**) 5-Sulfosalicylic acid, (**F**) Kynurenic acid, (**G**) Hippuric acid, (**H**) Indoleacetic acid, (**I**) Kynurenine, (**J**) Tryptophan, (**K**) Phenylacetic acid, and (**L**) *p*-cresol sulfate. Data are presented as mean ± SEM and statistical significance was assessed using one-way ANOVA followed by Tukey–Kramer post hoc test or Kruskal–Wallis test; *: $p < 0.05$; **: $p < 0.001$.

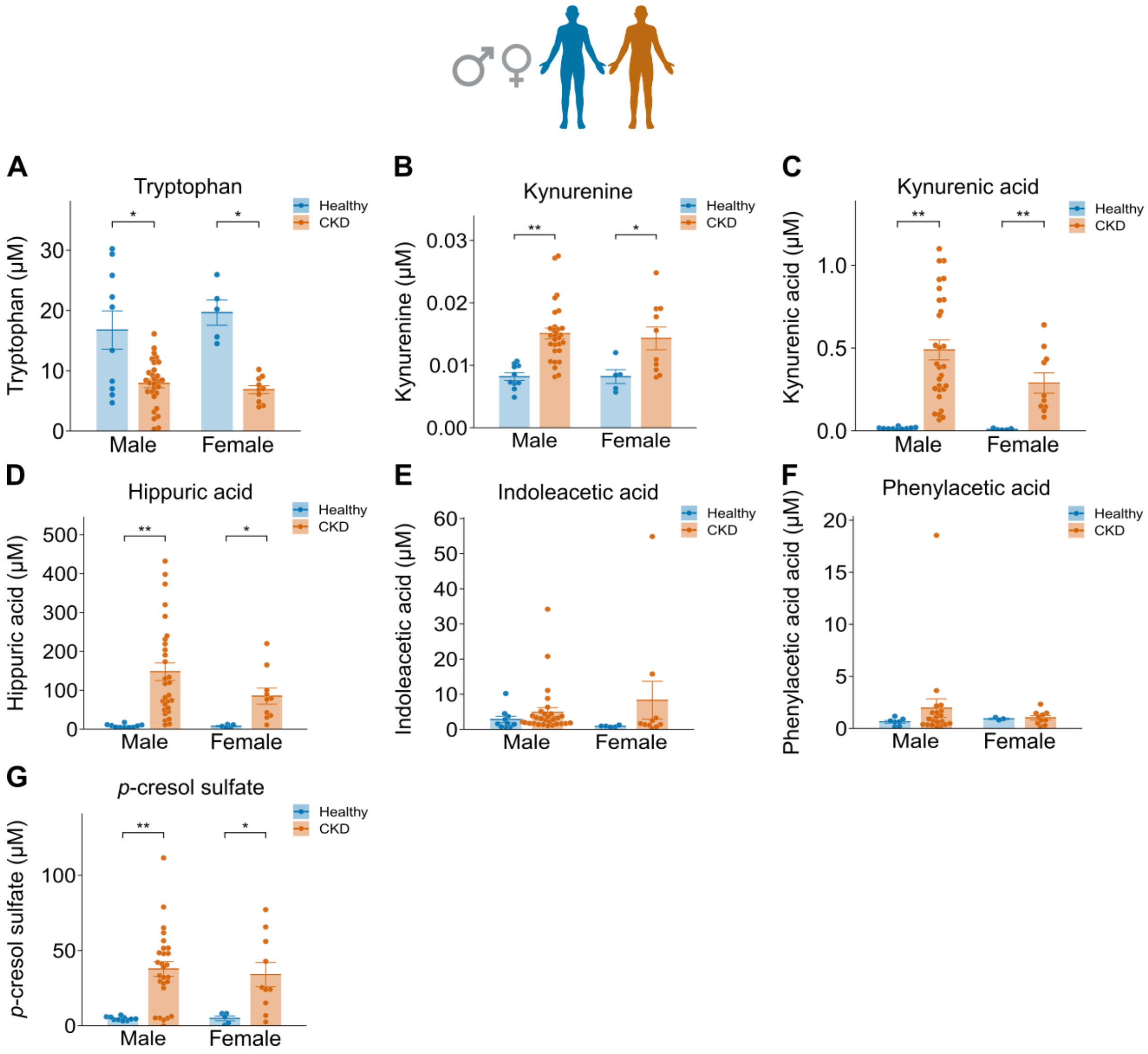

Supplemental Figure S5. Targeted plasma metabolomics from CKD patients. (**A**-**G**) Targeted quantification of plasma metabolites in human CKD patients (CKD men, *n*=29; CKD women, *n*=10,) and healthy controls (healthy men, *n*=10; healthy women, *n*=5). (**A**) Tryptophan, (**B**) Kynurenine, (**C**) Kynurenic acid, (**D**) Hippuric acid, (**E**) Indoleacetic acid, (**F**) Phenylacetic acid, and (**G**) *p*-cresol sulfate. Data are presented as mean ± SEM and statistical significance was assessed using one-way ANOVA followed by Tukey–Kramer post hoc test; *: $p < 0.05$; **: $p < 0.001$.

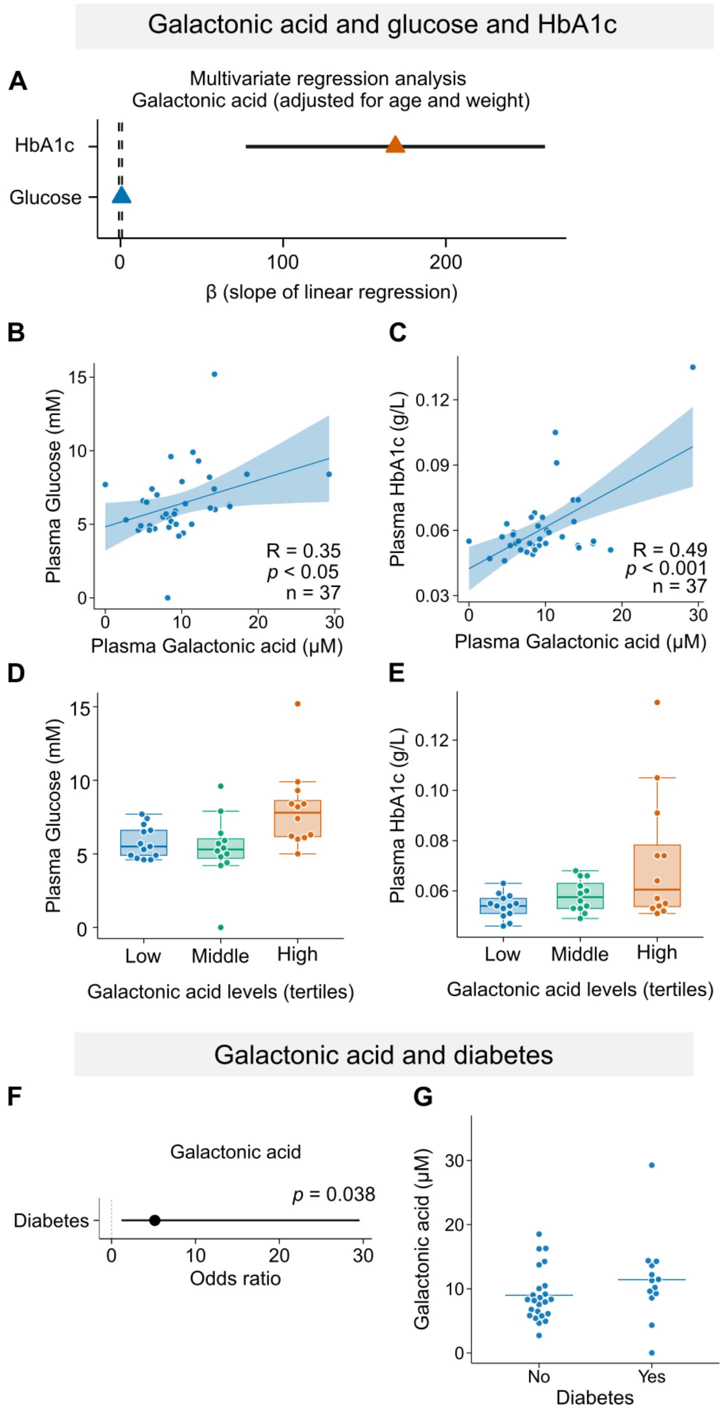

Supplemental Figure S6. Galactonic acid and diabetes**. (A)** Multivariate linear regression analyses of galactonic acid against HbA1c and glucose adjusted for age and body weight. Triangles indicate direction and significance of associations: blue triangles represent non-significant associations ($p \geq 0.05$), with upward- and downward-pointing symbols for positive and negative slopes, respectively; orange triangles represent significant associations ($p < 0.05$),again indicating directionality. β values and 95% confidence intervals are shown for each regression. (**B**-**C**) Linear regression analyses showing the relationship between plasma galactonic acid (**B**) plasma glucose and (**C**) plasma HbA1c. (**D**-**E**) plasma galactonic acid levels divided in tertiles from indicating low (*n*=15), middle (*n*=13) and high (*n*=13) levels of galactonic acid and the corresponding plasma concentrations of (**D**) glucose and (**E**) HbA1c. (**F**) The odds ratio for having diabetes between patients with galactonic acid levels below median compared with those above. The odds ratio is shown with 95% confidence intervals. (**G**) Galactonic acid concentrations stratified by diabetes status, either not having (No, *n*=24) or having diabetes (Yes, *n*=13).

Supplemental Table S1. Patient characteristics.

Basic characteristics	**Value (±SD)**
Patients (n)	39
Sex, male (%)	74.4
Age (years)	61 (14.3)
Height (cm)	173 (9.6)
Smoking, n (%)	28.21
Plasma potassium (mmol/L)	4.2 (0.7)
Plasma sodium (mmol/L)	138.4 (3.2)
Dialysis parameters	
Haemodialysis frequency (treatments/week)	2.82 (0.45)
Dialysis time (hours/week)	10.7 (3.0)
Dialysis vintage (days)	175 (102)
Duration of dialysis (hours)	3.77 (0.56)
Primary kidney disease	
Diabetic nephropathy, n (%)	28
Glomerulonephritis, n (%)	7.7
Hypertensive renal disease, n (%)	13
Kidney graft failure, n (%)	2.6
Other, n (%)	13
Polycystic kidney disease, n (%)	13
Unknown, n (%)	23
Kidney disease parameters	
Plasma creatinine (µmol/L)	620 (127)
Measured glomerular filtration rate (ml/min/m^2)	4.63 (3.16)
Plasma urea (mmol/L)	18.9 (5.73)
Hormones	
Plasma adrenaline (nmol/L)	0.11 (0.08)
Noradrenaline (nmol/L)	2.21 (1.3)
Plasma parathyroid hormone (pmol/L)	25.4 (24.2)
Cardiometabolic/diabetes parameters	
Plasma total cholesterol (mmol/L)	4.08 (0.92
Plasma phosphate (mmol/L)	1.63 (0.44)
Plasma glucose (mmol/L)	6.33 (2.32)
Plasma HDL (mmol/L)	1.13 (0.36)
Plasma LDL (mmol/L)	2.28 (0.81
HbA1c (g/L)	0.06 (0.02)
Plasma triglycerides (mmol/L)	1.53 (0.95)
Diabetes, n (%)	35.9
Cardiovascular parameters	
Diastolic blood pressure (mmHg)	74.4 (12.4)
Heart rate (dialysis end) (beats/min)	70.8 (21.8)
Heart rate (dialysis start) (beats/min)	73.1 (14.1)
Heart rate variability (HRV) mean N-N interval (ms)	689 (377)
Left ventricular ejection fraction (%)	61.7 (10.0)
Left ventricular end diastolic diameter (cm)	4.9 (0.56)

Left ventricular end systolic diameter (cm)	3.3 (0.59)
Left ventricular mass index (g/m^2)	120.9 (34.9)
Systolic blood pressure (mmHg)	144 (19.9)
Cardiovascular disease biomarkers	
NT-proBNP (mmol/L)	3.62 (4.6)
Troponin (nmol/L)	66.2 (63.8)
Inflammatory parameters	
CRP (mg/L)	4.19 (6.3)
IL-1β (pg/mL)	1.36 (1.27)
IL-6 (pg/mL)	16.9 (16.6)
IL-8 (pg/mL)	38.9 (34.2)
Cardiovascular disease outcomes	
Angina, n (%)	5
Arrhythmia, n (%)	13
Heart disease, n (%)	39
Heart valve disease, n (%)	5
Hypertension, n (%)	95
Ischemic heart disease, n (%)	26
Peripheral atherosclerosis, n (%)	23

Abbreviations: HDL, high-density lipoprotein; LDL, low-density lipoprotein; CRP, c-reactive protein; NT-proBNP, N-terminal pro b-type natriuretic peptide; HbA1c, hemoglobin A1c; IL, interleukin.

Supplemental Table S2. Primer sequences used for qPCR.

Target gene	Direction	Sequence (5′ to 3′)
Abcc4 (MRP4)	Sense	GAGATGGTGCAGAAGGGGAC
	Antisense	ACCAAATTGAGGCCTCGGAG
Abcg2 (BRCP)	Sense	TGGACTCAAGCACAGCGAAT
	Antisense	GGGTTGTTGTAGGGCTCACA
Acta2 (αSMA)	Sense	CTGACAGAGGCACCACTGAA
	Antisense	CATCTCCAGAGTCCAGCACA
Aqp1 (Aqp1)	Sense	GTCCAGGACAACGTGAAGGT
	Antisense	ACACACTGGGCGATGATGTA
Gapdh (GAPDH)	Sense	TAAAGGGCATCCTGGGCTACACT
	Antisense	TTACTCCTTGGAGGCCATGTAGG
Havcr1 (KIM1)	Sense	CGGTACAACTTAAAGGGGCA
	Antisense	GACGTGTGGGAATCTCTGGT
Slc22a2 (OCT2)	Sense	AAATGGTCTGCCTGGTCAAC
	Antisense	AGGCCAACCACAGCAAATAC
Slc22a6 (OAT1)	Sense	CTTCCTGTACACCGGAGAGC
	Antisense	AGCGCCGAAGATGAAGAGAG
Slc22a6 (OAT3)	Sense	TCTGGCCTGGTTTGCTACTG
	Antisense	CGCCGGCCCAGATAACTTAT
Slc47a1 (MATE1)	Sense	TCTTCAGACAGGACCCGGAT
	Antisense	GCGTTGACAAGGTTAGCTGC
Slco4c1 (OATP4c1)	Sense	ATCCCCGCCTTCTTCGAATC
	Antisense	TGCTAATGTTTACCAGGCCGT

Supplemental Table S3. Antibodies used for immunoblotting, immunohistochemistry, and immunofluorescence.

Target	Source	Dilution	Method	Manufacturer
Primary antibodies				
OCT2	Rabbit	1:250<br>1:500	Immunohistochemistry<br>Western blotting	ab230629; Abcam
OAT1	Rabbit<br>Rabbit	1:4,000<br>1:500	Immunohistochemistry<br>Western blotting	26574-1-AP; Proteintech<br>ab135924; Abcam
PDGFRβ	Rabbit	1:400	Immunofluorescence	ab32570; Abcam
αSMA	Mouse	1:500	Immunofluorescence<br>Western blotting	ab215368; Abcam<br>M0851; DAKO
AQP1	Rabbit	1:200	Immunofluorescence	AB2219; Sigma-Aldrich
KIM1	Goat	1:400	Immunofluorescence	AF1817; R&D Systems
Secondary antibodies				
Rabbit	Goat	1:2,000	Western blotting	P0488; DAKO
Rabbit	Goat	1:400	Immunohistochemistry	P0488; DAKO
Mouse	Goat	1:500	Immunofluorescence	Alexa Fluor 488; Invitrogen
Rabbit	Donkey	1:500	Immunofluorescence	Alexa Fluor 647; Invitrogen
Goat	Donkey	1:500	Immunofluorescence	Alexa Fluor 680; Invitrogen

Supplemental Table S4. Pure chemicals used as standards for targeted metabolomics.

Chemical	Manufacturer	Concentration range (μM)	ESI mode
L-Pipecolic acid	P2519, Sigma Aldrich, St. Louis, MI, USA	500 μM – 0.9 μM	Positive
D-Galactonic acid	S960098, Sigma Aldrich, St. Louis, MI, USA	500 μM – 2.1 μM	Negative
L-Kynurenic acid	K3375, Sigma Aldrich, St. Louis, MI, USA	100 μM – 0.14 μM	Positive
L-Kynurenine	K8625, Sigma Aldrich, St. Louis, MI, USA	400 nM – 0.5 nM	Positive
Hippuric acid	112003, Sigma Aldrich, St. Louis, MI, USA	500 μM – 0.7 μM	Positive
L-Tryptophan	T0254, Sigma Aldrich, St. Louis, MI, USA	300 μM – 0.4 μM	Positive
3-Indoleacetic acid	I2886, Sigma Aldrich, St. Louis, MI, USA	500 μM – 0.7 μM	Positive
Phenylacetic acid	P16621, Sigma Aldrich, St. Louis, MI, USA	450 μM – 0.6 μM	Negative
Indoxyl sulfate	I3875, Sigma Aldrich, St. Louis, MI, USA	100 μM – 15.2 nM	Negative
p-cresol sulfate	29504, Cayman Chemical, Ann Arbor, Michigan 48108, USA	450 μM – 0.6 μM	Negative
5-Sulfosalicylic acid	S2130, Sigma Aldrich, St. Louis, MI, USA	1000 μM – 20 nM	Negative
N-Acetylneuraminic acid	A2388, Sigma Aldrich, St. Louis, MI, USA	1000 μM – 20 nM	Negative

ESI: Electric spray ionization

Supplemental Table S5. Pipeline of the statistical methodology for identification of features in Compound Discover.

Node name	Processing step	Method description
Align Retention Times (ChromAlign)	Retention Time Alignment	Retention time alignment performed by the ChromAlign algorithm: Sadygov et al. (https://doi.org/10.1021/ac060923y).
Group Compounds	[Compounds] Apply Peak Rating Filter	Only compounds with an Original Peak Rating greater or equal to 3 in at least 5 samples are kept for further processing.
Fill Gaps	Similar Features Search	Features search within a tolerance of 5 ppm.
Fill Gaps	Centroids Filtering	Filtered centroids with S/N threshold = 1.5.
Fill Gaps	Detection	Real detection (for more accurate areas) was performed.
Apply SERRF QC Correction	QC Correction Method	QC correction performed by using SERRF method: Fan et al. (https://doi.org/10.1021/acs.analchem.8b05592).
Apply SERRF QC Correction	Random Forest Settings	Random Forest was run with 200 trees.
Apply SERRF QC Correction	Gap Filled Values	Gap filled areas from QC samples were used for QC.
Apply SERRF QC Correction	Interpolation	Values were interpolated with a non-linear regression.
Apply SERRF QC Correction	Correction Acceptance	The maximum QC RSD allowed before correction was set to 30%, and the maximum allowed after correction was set to 25%.
Apply SERRF QC Correction	Correction Acceptance	The minimum percentage of usable hits (actual signals found) per compound is 50%.
Differential Analysis	[Compounds] Input Data	Normalized Peak Area.
Differential Analysis	[Compounds] Group Area	Median.
Differential Analysis	[Compounds] Data Transformation	Log-10 areas for p-value estimation.
Differential Analysis	[Compounds] Statistical Test	The p-value of per group ratio calculated by a one-way ANOVA model with Tukey as post hoc test.
Differential Analysis	[Compounds] p-value Correction	p-value adjusted using Benjamini–Hochberg correction for the false-discovery rate.

Abbreviations: Ppm, parts per million; QC, quality control; RSD, relative standard deviation; S/N, Signal-to-noise.
